# High‐Performance Multi‐Dynamic Bond Cross‐Linked Hydrogel with Spatiotemporal siRNA Delivery for Gene–Cell Combination Therapy of Intervertebral Disc Degeneration

**DOI:** 10.1002/advs.202206306

**Published:** 2023-04-20

**Authors:** Jiaxin Chen, Haifeng Zhu, Jiechao Xia, Yutao Zhu, Chen Xia, Zehui Hu, Yang Jin, Ji Wang, Yong He, Jiayong Dai, Zhijun Hu

**Affiliations:** ^1^ Key Laboratory of Musculoskeletal System Degeneration Regeneration Translational Research of Zhejiang Province Department of Orthopaedic Surgery Sir Run Run Shaw Hospital Zhejiang University School of Medicine Hangzhou 310016 China; ^2^ Center for Plastic & Reconstructive Surgery Department of Plastic & Reconstructive Surgery Zhejiang Provincial People's Hospital Affiliated People's Hospital Hangzhou Medical College Hangzhou 310014 China; ^3^ Department of Orthopedic Surgery Zhejiang Provincial People's Hospital Affiliated People's Hospital Hangzhou Medical College Hangzhou 310014 China; ^4^ State Key Laboratory of Fluid Power and Mechatronic Systems College of Mechanical Engineering Zhejiang University Hangzhou 310027 China

**Keywords:** anti‐inflammatory, gene silencing, multi‐dynamic bonds, multifunctional hydrogels, tissue regeneration

## Abstract

Chronic inflammatory diseases, such as intervertebral disc degeneration (IVDD), which affect the lives of hundreds of millions of people, still lack effective and precise treatments. In this study, a novel hydrogel system with many extraordinary properties is developed for gene–cell combination therapy of IVDD. Phenylboronic acid‐modified G5 PAMAM (G5‐PBA) is first synthesized, and therapeutic siRNA silencing the expression of P65 mixed with G5‐PBA (siRNA@G5‐PBA) is then embedded into the hydrogel (siRNA@G5‐PBA@Gel) based on multi‐dynamic bonds including acyl hydrazone bonds, imine linkage, *π*–*π* stacking, and hydrogen bonding interactions. Local and acidic inflammatory microenvironment‐responsive gene‐drug release can achieve spatiotemporal regulation of gene expression. In addition, gene‐drug release from the hydrogel can be sustained for more than 28 days in vitro and in vivo, greatly inhibiting the secretion of inflammatory factors and the subsequent degeneration of nucleus pulposus (NP) cells induced by lipopolysaccharide (LPS). Through prolonged inhibition of the P65/NLRP3 signaling pathway, the siRNA@G5‐PBA@Gel is verified to relieve inflammatory storms, which can significantly enhance the regeneration of IVD when combined with cell therapy. Overall, this study proposes an innovative system for gene–cell combination therapy and a precise and minimally invasive treatment method for IVD regeneration.

## Graphical abstract

This work innovatively develops an injectable, self‐healing, adhesive, hemostatic, antibacterial, and biocompatible multifunctional hydrogel system for local and acidic inflammatory microenvironment‐responsive gene‐drug release and cell delivery, which is a precise and minimally invasive treatment method of IVDD and can achieve IVD regeneration through gene–cell combination therapy.

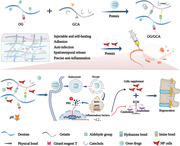

## 1 Introduction

Low back pain caused by intervertebral disc degeneration (IVDD) in middle‐aged and elderly people has become the leading cause of disability worldwide; it not only causes great pain to patients but also increases the burden on social healthcare.^[^ ^1^ ^]^ A normal intervertebral disc (IVD) consists of the central nucleus pulposus (NP), outer annulus fibrosus, and cartilage endplates connecting the adjacent vertebral bodies. The NP is the most important component of the IVD and is mainly composed of NP cells and the extracellular matrix (ECM). IVDD is characterized by a degraded microenvironment and unbalanced catabolic/anabolic activity of the NP.^[^ ^2^ ^]^ Studies have shown that the pathogenesis of IVDD involves a local inflammatory storm in which degenerative NP cells can overproduce a variety of pro‐inflammatory factors, such as IL‐6, IL‐1*β*, and TNF‐*α*, leading to the degradation of the ECM, thereby further aggravating the progression of disc degeneration.^[^ ^3^ ^]^ In addition, inflammation is also an important pathogenesis in the occurrence and development of chronic diseases of multiple other systems and organs, such as cancer, diabetes, cardiovascular disease, inflammatory bowel disease, and neurodegenerative disease,^[^ ^4^ ^]^ severely impacting the lives of numerous people. Therefore, finding an effective means to intervene in inflammation is crucial for the treatment of inflammation‐related diseases including IVDD and is of great significance to safeguarding public health.

Nuclear factor kappa‐light‐chain‐enhancer of activated B cells (NF‐*κ*B), a family of inducible transcription factors, are thought to be a key mediator in the regulation of various inflammatory diseases. NF‐*κ*B can directly induce the transcription of pro‐inflammatory cytokines, chemokines, and other inflammatory mediators in innate immune cells. In addition, the NF‐*κ*B signaling pathway is involved in mediating the activation of the NLRP3 inflammasome through binding to the promoter region of the NLRP3 gene, thus promoting its transcriptional expression and leading to the subsequent secretion of inflammatory factors and proptosis.^[^ ^5^ ^]^ Previous studies have revealed that P65/NLRP3 signaling pathway plays a crucial role in the inflammatory response during IVDD.^[^ ^6^ ^]^ Currently, several FDA‐approved drugs that can block NF‐*κ*B signaling have been developed, including aspirin, bortezomib, tacrolimus, imatinib mesylate, and dexamethasone.^[^ ^7^ ^]^ Nevertheless, considering that many problems associated with existing drugs remain, such as multi‐target effects, drug side effects, long treatment times, and drug resistance, it is still essential to explore new methods for precisely targeting the NF‐*κ*B signaling pathway to achieve safe and efficient regulation of the inflammatory microenvironment and the progression of inflammatory pathology.

Small interfering RNA (siRNA)‐related gene drugs have opened a new direction in drug development.^[^ ^8^ ^]^ By degrading the corresponding messenger RNA (mRNA) at the post‐transcriptional level, siRNA can markedly inhibit the expression of any specific and targeted gene.^[^ ^9^ ^]^ However, naked siRNA can easily be degraded by ribonuclease (RNase) in vivo and cannot pass through the cell membrane because of its negative charge, resulting in unsatisfactory transfection efficiency and therapeutic effect.^[^ ^10^ ^]^ Recently, phenylboronic acid (PBA)‐modified cationic polymers were reported as an attractive vehicle for gene delivery,^[^ ^11^ ^]^ PBA can induce strong binding of polymers to the cell surface by binding to plasma membrane glycoconjugates;^[^ ^12^ ^]^ moreover, through specific interactions with lysosomal membrane proteins and heat shock proteins, it can significantly enhance its lysosomal escape capacity.^[^ ^13^ ^]^ In addition, the chemical structure of boric acid can be designed to form reversible covalent borate ester bonds that dissociate in response to stimuli such as pH, reactive oxygen species (ROS), and glucose, enabling the controlled release of nanogene carriers in specific pathological settings.^[^ ^14^ ^]^ However, owing to the inherent limitations of RNAi technology, including the transient silencing effect, short half‐life, and abnormal distribution,^[^ ^15^ ^]^ there is an urgent need to develop biomaterials that support sustained local release and maintain the biological activity of siRNA.

Previous studies have used nanofiber‐mediated delivery systems to achieve long‐term siRNA delivery and target gene silencing,^[^ ^16^ ^]^ but these often required complex electrospinning techniques, and siRNA may lose its functional activity through interaction with organic solvents. Formed by cross‐linking of polymer networks under mild conditions, hydrogels with porous structures and good swelling properties, which can realize localized, prolonged, and sustained release of gene drugs in target tissues and organs, are considered to be an ideal delivery system.^[^ ^17^ ^]^ For example, a previous study utilized a covalent cross‐linking strategy that bound cholesterol‐modified sulfurized siRNA into photocrosslinked dextran to control and prolong the local release of siRNA through the hydrolysis of ester or disulfide bonds between the siRNA and hydrogel.^[^ ^18^ ^]^ Wang et al. developed a PEG‐based hydrogel in which siRNA nanocomplexes were embedded. Through localized and continuous WWP1 silencing, the hydrogel system exhibited the potential for accelerating bone formation.^[^ ^19^ ^]^ To date, little attention has been paid to the biologically multifunctional properties of hydrogels for siRNA delivery, greatly limiting the scope of their application in the field of biomedicine and clinical transformation. For instance, as the largest avascular tissue in the body, injectable drugs are especially suitable for the local treatment of IVDD because systemic administration fails to form effective drug concentrations in the inner NP tissue.^[^ ^20^ ^]^ Self‐healing characteristics can endow materials with the ability to adapt to the irregular shape of defects and withstand a certain amount of shear and tension.^[^ ^21^ ^]^ Antibacterial activity can effectively prevent implant‐related infections.^[^ ^22^ ^]^ Tissue adhesion and integration characteristics can allow for seamless scaffold–tissue matrix integration, which can provide stable biological fixation and achieve sustained release of drugs in the local space, thus promoting tissue regeneration.^[^ ^23^ ^]^ In addition, “smart” hydrogels that respond to biochemical cues of disease pathology can effectively schedule the release time of therapeutic drugs.^[^ ^24^ ^]^ Unfortunately, integrating all such functions and desirable properties into a single hydrogel system to realize accurate therapy for IVDD remains a huge challenge.

**Scheme 1:**
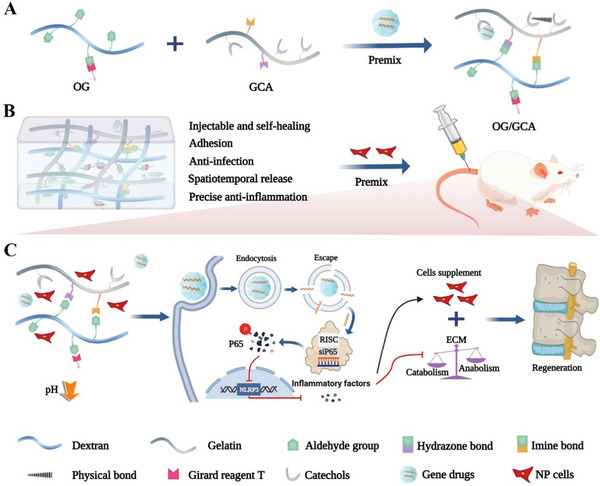
Schematic diagram of the synthetic route for the gene drug‐loaded multifunctional hydrogel and its gene–cell combination therapy application for IVD regeneration by precise targeting of the P65/NLRP3 signal pathway. A) Fabrication of the multi‐dynamic bond cross‐linked hydrogel for siRNA loading and delivery. B) In vivo rat IVDD minimally invasive therapy using both the gene‐drug and NP cell‐loaded multifunctional hydrogel. C) Underlying mechanism for IVD regeneration.

In this study, we design a novel in situ‐formed multifunctional hydrogel (OG/GCA) cross‐linked by multiple dynamic bonds, including acyl hydrazone bonds, imine linkage, *π*–*π* stacking, and hydrogen bonding interactions between two interpenetrating polymer chains, namely Girard reagent T‐modified oxidized dextran (OG) and adipic acid dihydrazide (ADH)‐grafted catechol‐coupled gelatin (GCA). In precursor strands of the gel, dextran is a natural polysaccharide with good biocompatibility and biodegradability,^[^ ^25^ ^]^ and as an ECM derivative, gelatin‐rich in RGD sequences can support cell adhesion, proliferation, and remodeling.^[^ ^26^ ^]^ The OG/GCA hydrogel possesses fast gelation, injectability, and self‐healing characteristics with potent adhesion, hemostasis, and antibacterial abilities. Before OG and GCA cross‐linking, siRNA@efficient transfection cationic gene carrier (PBA‐modified generation 5 poly (aminoamide) (PAMAM) dendrimer (G5‐PBA)) nanocomplexes are introduced into the hydrogel system. The borate group of PBA easily reacts with the catechol group in the GCA chain to form borate ester bonds,^[^ ^8^ ^]^ which can limit the rapid free diffusion of siRNA@G5‐PBA. The results demonstrate that the hydrogel could effectively control and sustain the release of siRNA for over 28 days in vitro and in vivo. Meanwhile, the embedded siRNA@G5‐PBA showed an acid‐responsive release property owing to the dissociation of the borate ester bond, acyl hydrazone bond, and imine bond when the pH value decreased,^[^ ^8^, ^27^ ^]^ which closely matched the acidic pathological microenvironment of IVDD.^[^ ^28^ ^]^ This novel hydrogel system can be injected locally and stably fixed on tissues (specific space) and exhibits a pH‐responsive release property (specific time) to achieve spatiotemporally controlled release of gene drugs and spatiotemporal regulation of cellular gene expression. Finally, by accurately restraining the P65/NLRP3 signaling pathways, the siP65@G5‐PBA@Gel system greatly inhibits the inflammatory cascade, thereby restoring the balance between the catabolism and anabolism of NP cells. Importantly, combined with NP cell co‐transplantation, gene therapy with the multifunctional hydrogel significantly enhanced IVD regeneration in a rat IVDD model (**Scheme** **1**).

## 2 Results and Discussion

### 2.1 Synthesis and Characterization of the PBA‐Modified Gene Carrier (G5‐PBA) for Efficient siRNA Transfection

**Figure 1:**
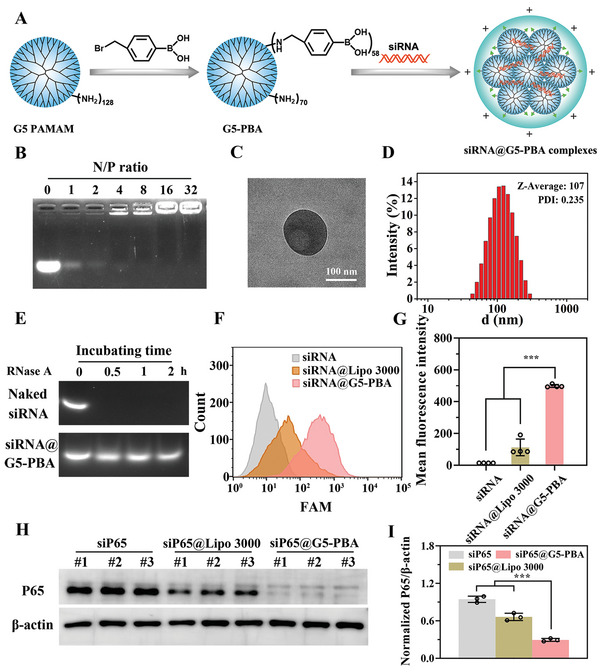
Characterization of G5‐PBA for siRNA delivery. A) Schematic illustration of the G5‐PBA synthesis and siRNA@G5‐PBA complex preparation. B) Agarose gel electrophoresis assay of siRNA@G5‐PBA complexes with different N/P ratios. C) TEM image of the optimized siRNA@G5‐PBA complexes (scale bar = 100 nm). D) Size distribution of siRNA@G5‐PBA. E) Agarose gel electrophoresis of complexes after incubation in RNase. F) Intracellular uptake of FAM‐siRNA@G5‐PBA in rat NP cells determined by flow cytometry. G) Mean fluorescence intensity calculated from the flow cytometry (*n* = 3). H) WB analysis of the silencing efficacy of the gene‐drug targeting the P65 protein on rat NP cells. I) Quantitative analysis of the WB images by Image J software (*n* = 3). The values presented are the mean ± SD. *** *p* < 0.001. Statistical significance was determined by one‐way ANOVA with a post‐hoc Tukey test for (G,I).

Small interfering RNA (siRNA)‐based gene therapy is a novel therapeutic approach that targets specific pathological pathways or genes for the precise treatment of related diseases.^[^ ^29^ ^]^ The gene vector (G5‐PBA) was synthesized by modifying PBA to a G5 PAMAM dendrimer using facile chemistry and then employed for siRNA loading and transfection. As shown in Figure S1, Supporting Information, proton nuclear magnetic resonance spectroscopy (^1^H NMR) confirmed that the PBA groups were successfully conjugated on the dendrimer surface, and the average number of PBA conjugates was calculated to be 58 based on the integrated areas. Different N/P ratios of siRNA@G5‐PBA complexes, which were calculated according to the ratio of the positively charged amino groups on the G5‐PBA surface to the negatively charged phosphate groups on the siRNA, were then prepared using a self‐assembly strategy (**Figure** **1**A). Agarose gel electrophoresis analysis indicated that G5‐PBA could stably bind to siRNA when the N/P ratio was greater than 4:1 (Figure 1B), and the zeta potential revealed that G5‐PBA changed the siRNA charge state from negative to positive at N/P ratios of greater than 2:1 (Figure S2A, Supporting Information). As the N/P ratio gradually increased, the efficiency of cellular uptake and gene transfection of siRNA@G5‐PBA in rat NP cells gradually increased, accompanied by increased cytotoxicity (Figure S2B,C, Supporting Information). In view of the balance between high transfection efficacy and low cytotoxicity, an N/P ratio of 16:1 was chosen as the optimal condition and used for subsequent studies. Transmission electron microscopy (TEM) revealed a typical nanoparticle microstructure of the siRNA@G5‐PBA complexes (Figure 1C); the diameter of the complexes was ≈100 nm (Figure 1D). In addition, as shown in Figure 1E, G5‐PBA could effectively stabilize siRNA in the presence of 0.1 mg mL^−1^ RNase after 2 h. Naked siRNA was rapidly degraded, indicating the RNase resistance ability of siRNA@G5‐PBA. To further evaluate the transfection efficacy of the complexes at an N/P ratio of 16:1, the commercial transfection reagent Lipo 3000 was introduced as a positive control, and the results revealed that the mean fluorescence intensity (FAM‐labeled siRNA) of cells transfected by G5‐PBA was stronger than that of cells transfected with Lipo 3000 (Figure S2D, Supporting Information and Figure 1F,G).

The silencing efficacy of target genes is most critical for gene delivery materials; thus, siRNA nanocomplexes targeting the key inflammatory targets (P65 gene) were constructed and transfected into NP cells. P65, known as RelA, is one of the five components comprising the NF‐*κ*B family of transcription factors. Inflammation or other related gene transcription is generally induced through the P65 signal pathway under various stimuli.^[^ ^7^ ^]^ Previous studies have also shown that the phosphorylated P65 (P‐P65) subunit plays a key role as a marker of NF‐*κ*B activation during IVDD progression.^[^ ^30^ ^]^ Western blot (WB) analysis demonstrated that G5‐PBA was able to more effectively and stably knock down the expression of the P65 protein in rat NP cells compared to the group treated with Lipo 3000 (Figure 1H,I). This may be attributed to the powerful combination between the cell membrane and boric acid group in G5‐PBA^[^ ^12^ ^]^ as well as the excellent endosomal escape ability of PBA^[^ ^13^ ^]^ and protonable tertiary amine groups in the dendrimer interior.^[^ ^31^ ^]^ Taken together, these results indicate that the gene delivery vector G5‐PBA, which was able to condense siRNA into positively charged nano‐sized complexes, stabilize the bound siRNA, and exhibit low cytotoxicity and efficient transfection efficacy, is a promising candidate for gene delivery.

### 2.2 Preparation and Characterization of the OG and GCA

OG was synthesized by NaIO_4_ oxidation and the Schiff base reaction (Figure S3A, Supporting Information). Under strong oxidation, dialdehyde groups were modified onto a dextran (DEX) backbone. This transformation was confirmed by a new peak at 1734 cm^−1^ in the Fourier transform infrared (FTIR) spectrum and a new proton peak at 9.6 ppm in the ^1^H NMR spectrum of oxidized dextran (ODEX) compared with those of DEX (Figure S3B,C, Supporting Information).^[^ ^32^ ^]^ Then, through the Schiff base reaction between amino groups in Girard reagent T and aldehyde groups in ODEX, OG was successfully synthesized, as confirmed by ^1^H NMR (Figure S3D, Supporting Information). GCA was obtained by coupling catechols onto a gelatin chain (GC) via EDC/NHS chemistry and further grafting ADH onto the GC backbone via EDC/HOBt chemistry, as confirmed by ^1^H NMR (Figure S4A,B, Supporting Information). As shown in Figure S5A, Supporting Information, compared with gelatin, GC exhibited stronger absorption at around 280 nm in the UV–vis spectra, also implying the successful modification of catechols; the degree of substitution was calculated to be 3.1% according to the established standard curve of catechols, as shown in Figure S5B, Supporting Information.^[^ ^33^ ^]^ ADH grafting was also verified by the UV–vis spectra. The typical absorption peak at around 505 nm was attributed to the reaction between ADH and TNBS, and the degree of ADH functionalization was calculated to be 4.7% (Figure S5C,D, Supporting Information).^[^ ^34^ ^]^ These results show that OG and GCA were successfully fabricated.

### 2.3 Design, Preparation, and Characterization of the OG/GCA Hydrogel

**Figure 2:**
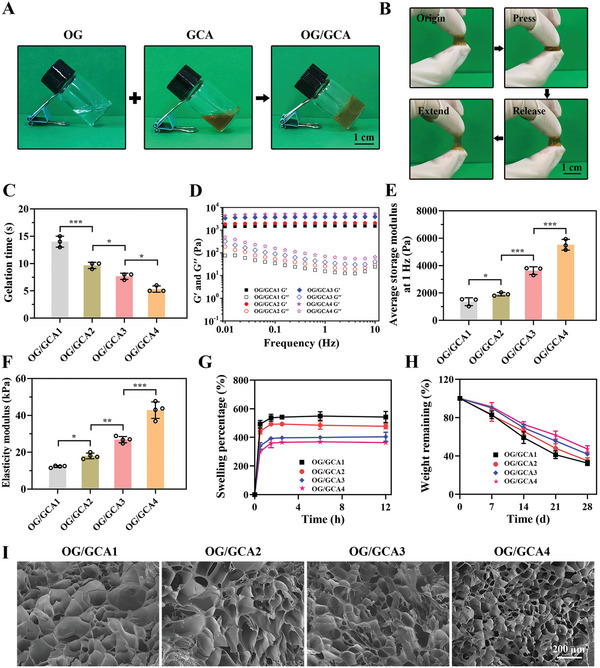
Characterizations of the OG/GCA hydrogels. A) Photographs of the OG solution, GCA solution, and multiple dynamic bond cross‐linked OG/GCA hydrogels (scale bar = 1 cm). B) Photographs showing the compressible and malleable nature of the hydrogels (scale bar = 1 cm). C) Gelation times obtained from vial‐tilting tests of hydrogels with different polymer concentrations (*n* = 3). D) Frequency sweep tests (from 0.01 to 10 Hz) at 37 °C of the hydrogels with varying concentrations. E) Quantitative average storage modulus at 1 Hz of the hydrogels determined by frequency sweep tests (*n* = 3). F) Elasticity modulus of the hydrogels in the 10–20% strain range of the compression curves (*n* = 4). G) Swelling property of the hydrogels in PBS at 37 °C (*n* = 3). H) In vitro degradation property of the hydrogels obtained by evaluating the weight remaining in PBS at 37 °C under shaking at 100 rpm (*n* = 3). I) SEM images of the hydrogels (scale bar = 200 µm). OG/GCA1: 5% OG and 10% GCA; OG/GCA2: 8.5% OG and 10% GCA; OG/GCA3: 8.5% OG and 15% GCA; OG/GCA4: 12% OG and 15% GCA. OG/GCA3 was used for photographs. The values presented are the mean ± SD. * *p* < 0.05; ** *p* < 0.01; *** *p* < 0.001. Statistical significance was assessed by one‐way ANOVA with a post‐hoc Tukey test for (C, E,F).

The synthesized OG and GCA were used as precursor polymers, which were then mixed in equal volumes at room temperature to form the hydrogel through multi‐dynamic bond cross‐linking, including acyl hydrazone bonds, imine linkages, *π*–*π* stacking, and hydrogen bonds, without adding any toxic cross‐linkers (**Figure** **2**A). As shown in Figure 2B, the hydrogel could withstand a certain degree of compression and stretching. Then, hydrogels of different concentrations were prepared, and the gelation time was measured using a vial‐tilting test. The results showed that gelation occurred in less than 20 s in all groups, and the rate accelerated as the concentration of OG or GCA increased (Figure 2C). The rapid gelation time of the hydrogel could be attributed to the introduction of the amino groups of ADH, which contribute to fast gene‐drug encapsulation and effective loss reduction during encapsulation. The mechanical properties are of great significance for the application of hydrogels as biomaterials, and these were evaluated using rheological and compressive tests. The frequency sweep results showed that the storage modulus (G′) remained almost constant throughout and was much higher than the loss modulus (G″) when the frequency increased from 0.01 to 10 Hz, revealing the viscoelastic properties of the hydrogels and long‐term network stability in the range of the natural IVD loading frequency (≈4–5 Hz, Figure 2D).^[^ ^35^ ^]^ In addition, with an increase in polymer concentration, the average G′ at 1 Hz increased (Figure 2E). The compression analysis results were consistent with the rheology: the elasticity modulus of the hydrogels calculated in the strain range of 10–20% was improved with increasing concentration, indicating improved mechanical strength due to the increased crosslinking density (Figure S6, Supporting Information and Figure 2F). The swelling behavior of the hydrogels is shown in Figure 2G, where all of the hydrogel groups swelled rapidly, and the swelling percentage decreased with increasing cross‐linking density. The degradation rate of the hydrogels under physiological conditions in vitro was estimated by placing them in PBS at 37 °C at a shaking speed of 100 rpm. With the extension of days, the hydrogels gradually degraded because of the broken of the multi‐dynamic bonds (Figure 2H). Besides, we can see that there was still hydrogel residue over 4 weeks which should be attributed to the relatively stable hydrazone bond cross‐linking;^[^ ^36^ ^]^ this stability is a desirable trait for long‐term gene‐drug delivery and in vivo applications. The representative porous structure of the hydrogel was observed using scanning electron microscopy (SEM), and an increased cross‐linking density led to a decrease in aperture (Figure 2I and Figure S7, Supporting Information). These results reflect the adjustable physical characteristics of the as‐designed hydrogel system and its ideal properties for scaffold or vector applications.

### 2.4 Adhesive, Hemostatic, and Antibacterial Performance of the OG/GCA Hydrogel

**Figure 3:**
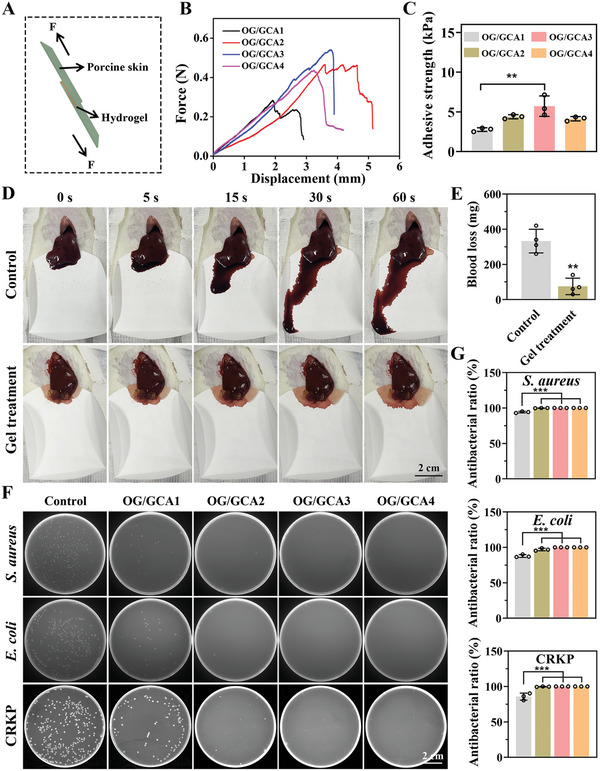
Adhesive, hemostatic, and antibacterial properties of the OG/GCA hydrogel. A) Schematic diagram of the lap shear test using porcine skin. B) Typical force‐displacement curves of porcine skin bonded to hydrogels with varying concentrations. C) Quantitative adhesive strength of the hydrogels calculated from the force‐displacement curves (*n* = 3). D) Photographs of blood loss in the liver after treatment with the hydrogel (scale bar = 2 cm). E) Quantitative blood loss analysis (*n* = 4). F) Photographs of bacterial colonies formed by *S. aureus*, *E. coli*, and CRKP after treatment with different hydrogels (scale bar = 2 cm). G) Quantitative antibacterial rate analysis (*n* = 3). OG/GCA1: 5% OG and 10% GCA; OG/GCA2: 8.5% OG and 10% GCA; OG/GCA3: 8.5% OG and 15% GCA; OG/GCA4: 12% OG and 15% GCA. OG/GCA3 was used for the in vivo bleeding experiment. The values presented are the mean ± SD. ** *p* < 0.01; *** *p* < 0.001. Statistical significance was determined by one‐way ANOVA with a post‐hoc Tukey test for (C,G), and by a two‐tailed Student's *t*‐test for (E).

Its wet tissue adhesion properties make the hydrogel suitable for repairing highly dynamic tissue defects, localizing and releasing loaded drugs in a specific space, and promoting material–tissue integration.^[^ ^23a^ ^]^ The adhesion ability of the hydrogel to various wet tissues was macroscopically observed, as shown in Figure S8A, Supporting Information, The OG/GCA hydrogel could tightly adhere to two pieces of heart, liver, spleen, lung, kidney, and IVDs without any external assistance. Additionally, the hydrogel could adhere to porcine skins intact and tightly upon underwater placement and running water flushing (Figure S8B,C and Video S1, Supporting Information), which was attributed to multiple covalent and non‐covalent cross‐links (based on aldehyde and catechol groups) between the hydrogels and tissue surfaces. Then, lap shear measurements on fresh porcine skin were performed to quantify the wet tissue adhesive strength of the hydrogels (**Figure** **3**A). As shown in Figure 3B,C, the OG/GCA1, OG/GCA2, OG/GCA3, and OG/GCA4 hydrogels exhibited adhesive strengths of 2.8, 4.4, 5.7, and 4.1 kPa, respectively. Although the concentrations of OG and GCA in the OG/GCA4 hydrogel were the highest, we propose that more groups used for cross‐linking of the internal dense network may account for its reduced adhesion strength. These results verify that the hydrogel exhibits good adhesion and tissue‐integration properties.

Although a minimally invasive operation was performed in this study, timely stanching of bleeding is indispensable. Therefore, a rat liver bleeding model was established to evaluate the hemostasis ability of the hydrogels. As shown in Figure 3D and Video S2, Supporting Information, the hydrogel could adhere to the hemorrhage sites and immediately stop the bleeding. Quantitative analysis indicated that the total blood loss in the untreated group was 332.5 mg, compared with 75.0 mg in the hydrogel‐treated group (Figure 3E), confirming that the increased covalent and non‐covalent bond interaction between hydrogels and tissues due to the aldehyde and catechol groups could form a protective barrier for hemostasis. Furthermore, gelatin could also simultaneously activate and aggregate platelets and accelerate hemostasis.^[^ ^37^ ^]^

Implant material‐related infections that impair tissue healing should not be ignored. Antibiotics are widely used in the field of antibacterial therapy; however, there is a potential risk of drug resistance.^[^ ^38^ ^]^ Cationization of the polymer with Girard reagent T, which can electrostatically interact with the negatively charged bacterial cell membranes and effectively inactivate pathogens, could endow the hydrogel with antibacterial properties. As presented in Figure 3F, all of the OG/GCA hydrogel groups inhibited the growth of *Staphylococcus aureus*, *Escherichia coli*, and carbapenem‐resistant *Klebsiella pneumoniae* (CRKP) on plates. The antibacterial efficacies of the OG/GCA2, 3, and 4 hydrogels to *S. aureus*, *E. coli*, and CRKP bacteria were all greater than 99% (Figure 3G), indicating broad‐spectrum antibacterial activity of the hydrogels. To further investigate the antibacterial mechanism of the OG/GCA hydrogel, SEM characterization was performed. As shown in Figure S9, Supporting Information, the morphology of the *S. aureus*, *E. coli*, and CRKP bacteria in the control group was full with smooth and intact bacterial cell membranes, whereas the bacteria treated with the hydrogel exhibited wrinkled and damaged bacterial cell membranes. These results confirm that the bacterial cell walls were severely damaged by the hydrogel owing to the Girard reagent T functionalization, which contributed to the subsequent disruption of the integrity of the bacterial cells and the eventual death of the bacteria. Considering the physical properties, adhesive strength, and antibacterial activity of the hydrogel, OG/GCA3 was selected for the subsequent experiments.

### 2.5 Injectable and Self‐Healing Properties of the Hydrogel

**Figure 4:**
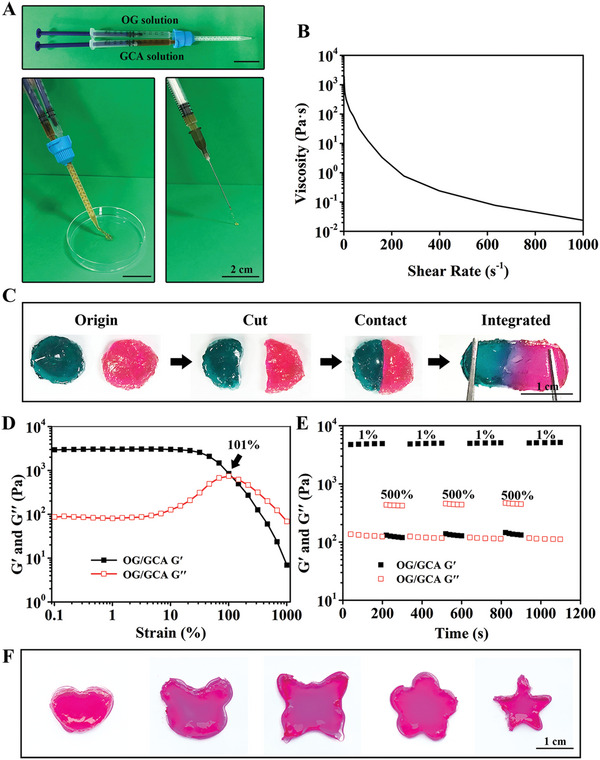
Injectable and self‐healing properties of the OG/GCA hydrogel. A) Photographs of the hydrogel injected with a dual‐channel injector and 21G needle (scale bar = 2 cm). B) Viscosity measurements of the hydrogel with increasing shear rate (0.1–1000 s^−1^) at a fixed frequency of 1 Hz at 37 °C. C) Photographs of the macro self‐healing process of the hydrogel dyed with rhodamine B and methylene blue (scale bar = 1 cm). D) Rheological properties of the hydrogel in the strain amplitude sweep (*γ* = 0.1–1000%) at 1 Hz and 37 °C. E) Rheological properties of the hydrogel at low (1%) and high (500%) strains with loading times of 200 and 100 s, respectively. F) Photographs of the shape adaptability of the hydrogel (scale bar = 1 cm).

Injectable hydrogels have unique advantages for local minimally invasive therapeutic applications. After injecting the precursor using a dual‐channel injector, the two solutions were mixed, and the gelation process occurred immediately (**Figure** **4**A). The prefabricated hydrogel could be continuously injected through a 21G needle without any clogging. The shear viscosity curve showed that the viscosity decreased rapidly with increasing shear rate, revealing the shear‐thinning behavior of the hydrogel (Figure 4B). Traditional covalently cross‐linked hydrogels are prone to become worn or even damaged under external mechanical forces and cannot repair themselves. The OG/GCA hydrogel based on multi‐dynamic bonds was well equipped with self‐healing properties, greatly prolonging its service life, allowing it to adapt to irregular shapes, and preventing pressure‐induced breakdown.^[^ ^39^ ^]^ As shown in Figure 4C, rhodamine B‐dyed and methylene blue‐dyed hydrogels were cut into two halves and then combined. Interestingly, the divided hydrogel could be restored to an intact piece after 30 min. To further assess the self‐healing properties of the hydrogel, strain amplitude sweep measurements were conducted. Figure 4D shows that the G′ and G′′ curves intersected at a strain of 101%. G′ became lower than G′′ when the strain was larger than the critical point, suggesting a probable collapse of the hydrogel network and the transition from gel to sol.

Thereafter, the recoverability of the hydrogel was evaluated using a continuous step‐strain test with alternating strains of 1% and 500%. Under the higher strain (500%), the hydrogel structure collapsed (G′′ > G′). When a lower strain (1%) was then applied, the hydrogel immediately returned to its original state (G′ > G′′) with almost the same modulus as the original hydrogel. The collapse and recovery of the OG/GCA hydrogel network were reversible and could be maintained over three repeated cycles (Figure 4E), demonstrating the rapid and excellent self‐healing ability of this hydrogel based on the reversible breaking and re‐formation of the multi‐dynamic bonds. Moreover, it can be clearly observed from Figure 4F that the hydrogel can be molded into any desired shape, indicating the potential of the hydrogel to fill irregular tissue defects.

### 2.6 Biocompatibility of the siRNA@G5‐PBA@Gel

**Figure 5:**
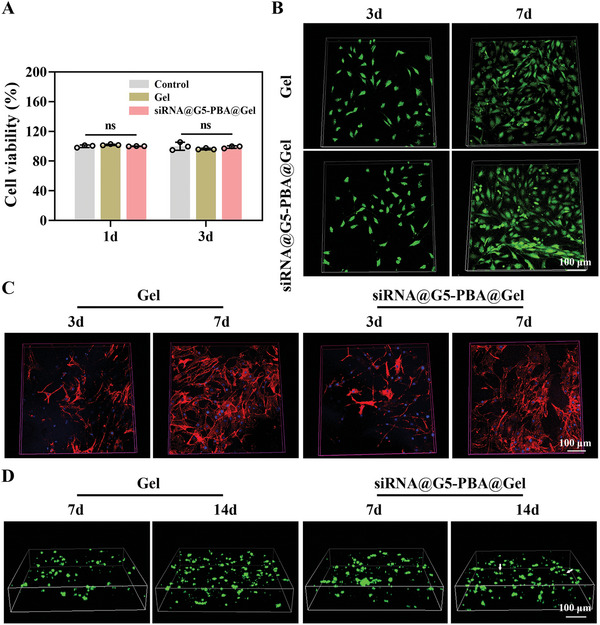
Cytocompatibility of the siRNA@G5‐PBA@Gel. A) Viability determined by CCK‐8 assay of rat NP cells after co‐cultivation with the complex‐loaded multifunctional hydrogel for 1 and 3 days (*n* = 3). B) Live/dead staining of NP cells cultured on the surface of hydrogels for 3 and 7 days (scale bar = 100 µm). C) Phalloidin staining of NP cells cultured on the surface of hydrogels for 3 and 7 days (scale bar = 100 µm). D) Live/dead staining of the encapsulated rat NP cells in the Gel or siRNA@G5‐PBA@Gel after 7 and 14 days (scale bar = 100 µm). Dead cells stained with red color are indicated using white arrows. The values presented are the mean ± SD. ns indicates not statistically significant. Statistical significance was assessed by one‐way ANOVA with a post‐hoc Tukey test for (A).

After the development of the multifunctional hydrogel, the gene‐drug siRNA@G5‐PBA was mixed with the precursor polymer chain to form gene‐drug‐loaded hydrogels (siRNA@G5‐PBA@Gel), and the biocompatibility was investigated. The CCK‐8 assay showed that the viability of NP cells co‐cultured with the Gel or siRNA@G5‐PBA@Gel was over 95% after 1 and 3 days of incubation (**Figure** **5**A). Live/dead staining showed that NP cells plated on the surface of the Gel or siRNA@G5‐PBA@Gel groups maintained high activity (Figure 5B). In addition, phalloidin was used to detect the cytoskeleton of NP cells plated on the hydrogel surface. As shown in Figure 5C, a large number of cells presented fusiform shapes after 3 and 7 days of cultivation, which was attributed to the abundant RGD sequences in the natural gelatin chain.^[^ ^26^ ^]^ In Figure 5D, the encapsulated rat NP cells in the siRNA@G5‐PBA@Gel also exhibited a certain proliferative capacity and a high level of vitality after 7 and 14 days of 3D cultivation. These results indicate the nontoxicity and good cytocompatibility of the gene drug‐loaded hydrogel system, which is an essential requirement for in vivo applications of biomedical materials.

A hemolysis assay was conducted to estimate the hemocompatibility of the hydrogels. Triton X‐100 was used as the positive control group, and normal saline was used as the negative control group. As shown in Figure S10A, Supporting Information, the color in both the Gel and siRNA@G5‐PBA@Gel group was similar to the normal saline group, while a significant difference with the Triton X‐100 treated group was observed. The quantitative hemolysis ratio of the siRNA@G5‐PBA@Gel group was less than 5% (Figure S10B, Supporting Information), implying excellent hemocompatibility of the gene drug‐embedded hydrogels.

The Gel and siRNA@G5‐PBA@Gel were then injected subcutaneously into rats to further investigate the histocompatibility of the hydrogels (Figure S11A, Supporting Information). We can see from Figure S11B, Supporting Information that both the Gel and siRNA@G5‐PBA@Gel hydrogels degraded gradually over time. They were almost completely degraded after 8 weeks, demonstrating the biodegradability of the hydrogels in vivo. In addition, no apparent tissue damage or abscesses were observed at the subcutaneous injection sites. Hematoxylin and eosin (H&E) staining also demonstrated no obvious inflammatory response in the surrounding skin tissues after 1, 2, 4, and 8 weeks of implantation (Figure S11C, Supporting Information). Furthermore, H&E staining of primary organs such as the heart, liver, spleen, lung, and kidney after 8 weeks of implantation revealed no toxic side effects from the hydrogels or their degradation products, and the liver, kidney function, and hematologic indices in the Gel and siRNA@G5‐PBA@Gel groups were all within normal ranges and similar to those in the non‐implantation group (Figure S12, Supporting Information). Therefore, the siRNA@G5‐PBA@Gel demonstrates outstanding cytocompatibility, hemocompatibility, and histocompatibility and is applicable for further in vitro and in vivo efficacy studies.

### 2.7 In Vitro and in Vivo Gene Drug Release from the Multifunctional Hydrogel

**Figure 6:**
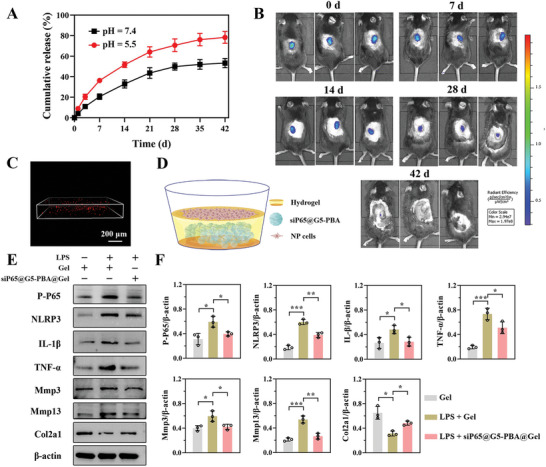
In vitro and in vivo siRNA@G5‐PBA release from the multifunctional hydrogel and the therapeutic effect of the siP65@G5‐PBA@Gel on NP cells. A) Cumulative release curve of Cy5‐siRNA@G5‐PBA from the hydrogel in PBS at pH 5.5 and 7.4 (*n* = 3). B) Real‐time fluorescence imaging of the Cy5‐siP65@G5‐PBA‐loaded hydrogel after subcutaneous injection into the back of mice. C) Fluorescence observation of Cy5‐siP65@G5‐PBA after embedding in the hydrogel (scale bar = 200 µm). D) Schematic representation of rat NP cells seeded on the surface of the siP65@G5‐PBA@Gel. E) Representative Western blot images of P‐P65, NLRP3, IL‐1*β*, TNF‐*α*, Mmp3, Mmp13, and Col2a1 protein expression in NP cells. F) Quantitative analysis of the WB images by Image J (*n* = 3). The values presented are the mean ± SD. * *p* < 0.05; ** *p* < 0.01; *** *p* < 0.001. Statistical significance was assessed by one‐way ANOVA with a post‐hoc Tukey test for (F).

Cy5‐labeled siRNA was used to evaluate the responsive release behavior of the siRNA@G5‐PBA nanocomplexes from the OG/GCA hydrogel in vitro and in vivo. Cy5‐siRNA@G5‐PBA@Gel was incubated in PBS (pH = 5.5 and 7.4) at a shaking speed of 100 rpm at 37 °C. Sustained release of the gene‐drug from the hydrogel could be maintained for over 28 days at pH values of both 5.5 and 7.4 in PBS (**Figure** **6**A). As shown in Figure 6B, real‐time fluorescence imaging of the Cy5‐siRNA@G5‐PBA‐loaded hydrogel was carried out through back subcutaneous injection, and the results confirmed that the gene‐drug could remain in the local site for over 28 days. These results demonstrated that the goal of the sustained local release was successfully realized owing to the interaction between siRNA@G5‐PBA and the hydrogel, particularly the formation of borate ester bonds. In addition, Figure 6A also showed that the gene‐drug was released in greater quantities and more rapidly in PBS at pH = 5.5 compared with the release performance at pH = 7.4, which verified the pH‐responsive release property of the siRNA@G5‐PBA@Gel. This is in agreement with the acid‐sensitive properties of the acyl hydrazone bonds, imine bonds, and borate ester bonds in the hydrogel system.^[^ ^8^, ^27^ ^]^ Owing to the acidic pathological microenvironment of IVDD,^[^ ^28^ ^]^ the acid‐responsive characteristic of this hydrogel has great potential to be employed to achieve efficient and precise gene therapy by releasing gene drugs at a specific time.

### 2.8 Therapeutic Assessment of the siP65@G5‐PBA@Gel in Vitro

Next, siRNA targeting of the P65 protein was obtained. After encapsulation in the OG/GCA hydrogel, the red fluorescence of Cy5‐labeled siP65@G5‐PBA could be clearly observed by confocal microscopy (Figure 6C), confirming the successful encapsulation of the gene drug. To further investigate the biological activity of the sustained‐release of gene‐drug, NP cells were directly seeded on the surface of the hydrogel (Figure 6D) and cultivated for 3 days, lipopolysaccharide (LPS) was then used to induce an inflammatory response and subsequent imbalance of the ECM catabolism and anabolism. From the western blot images and semiquantitative analysis shown in Figure 6E,F, we could see that the expressions of P‐P65, NLRP3, IL‐1*β*, and TNF‐*α* induced by LPS were significantly inhibited by the gene drug‐encapsulated hydrogel. In addition, the siP65@G5‐PBA@Gel could down‐regulate the expression of catabolic proteins such as Mmp3 and Mmp13 and rescue the content of ECM, as evidenced by a higher expression of Col2a1 protein. These results reveal that by precisely silencing the P65/NLRP3 signaling pathway, siP65@G5‐PBA@Gel could inhibit inflammatory storms and consequently recover the balance of ECM catabolism and anabolism, thus delaying the degeneration of NP cells.

### 2.9 Evaluation of IVD Regeneration in Vivo by siP65@G5‐PBA@Gel Combined with Cell Therapy

The gene drug‐loaded hydrogel, which precisely targets the NF‐*κ*B signaling pathway, was demonstrated above to significantly inhibit IVD inflammation and degeneration in vitro; however, it still lacks the ability of regeneration. Several previous studies have indicated that cell therapy is a promising treatment for promoting IVD repair and regeneration. However, several obstacles, such as low cell retention at local sites, poor cell survival, and differentiation directly exposed to the difficult inflammatory microenvironment, have significantly limited cell therapy applications. Simple cellular implantation may lead to a low number of integrated cells, significantly hindering the therapeutic effect and constituting a major challenge for cell therapy.^[^ ^40^ ^]^ The adhesive and local inflammation administration multifunctional hydrogel system developed in this study can provide a highly biocompatible platform for the minimally invasive delivery of cells. Therefore, the effect of OG/GCA hydrogel co‐delivery of a gene‐drug and NP cells for IVD regeneration in vivo was investigated.

**Figure 7:**
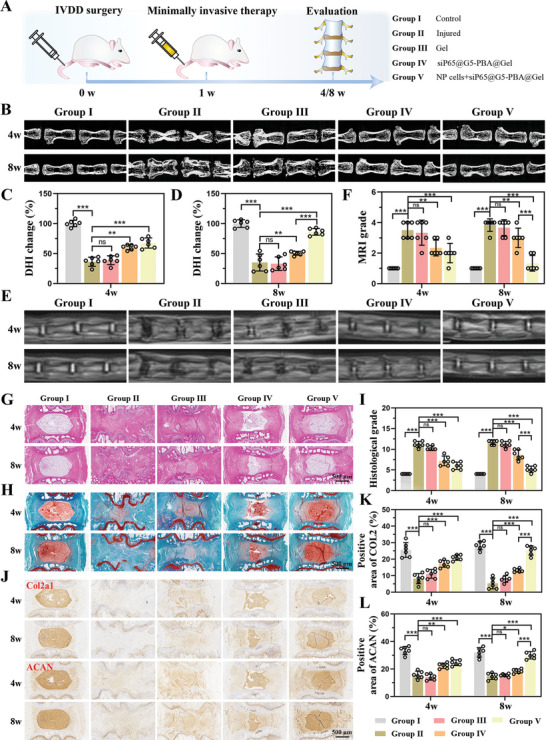
In vivo evaluation of IVD regeneration by siP65@G5‐PBA@Gel combined with cell therapy. A) Schematic illustration showing the overall procedure of the in vivo experiment. B) Micro‐CT images of rat coccygeal vertebrae discs in different groups at 4 and 8 weeks postoperative. Quantitative DHI changes according to micro‐CT images at C) 4 weeks (*n* = 6 discs) and D) 8 weeks (*n* = 6 discs) postoperative. E) MRI images and F) quantitative MRI grade of IVDs in different groups at 4 and 8 weeks postoperative (*n* = 6 discs). G,H) H&E, safranin O, and fast green staining of IVDs in rats at 4 and 8 weeks postoperative (scale bar = 500 µm). I) Changes in the histological grades at 4 and 8 weeks postoperative (*n* = 6 discs). J) Immunohistochemistry images of Col2a1 and ACAN at 4 and 8 weeks postoperative (scale bar = 500 µm). K,L) Quantitative positive areas of Col2a1 and ACAN analyzed by Image J (*n* = 6 discs). The values presented are the mean ± SD. * *p* < 0.05; ** *p* < 0.01; *** *p* < 0.001; ns, not statistically significant. Statistical significance was assessed by one‐way ANOVA with a post‐hoc Tukey test for (C,D,K,L), and by the Kruskal–Wallis H test for (F,I).

A needle puncture‐induced IVDD model in rats was established, followed by minimally invasive injection treatment, as described in a number of previous studies.^[^ ^20^, ^21^, ^41^ ^]^ Five groups were established, including the control group receiving no needle puncture (group I), the injured group receiving a puncture without injection (group II), the gel injection group (group III), the siP65@G5‐PBA@Gel injection group (group IV), and the siP65@G5‐PBA@Gel combined with NP cell injection group (group V, **Figure** **7**A). Micro‐CT images revealed that at 4 weeks postoperative, the disc height index (DHI%) in the siP65@G5‐PBA@Gel combined with NP cell group was similar to that of the siP65@G5‐PBA@Gel group, which were significantly greater than that of the injured and gel groups. The DHI% values in the NP cell co‐delivery group (group V) were better and similar to those in the control group, while those in the siP65@G5‐PBA@Gel declined at 8 weeks postoperatively. In addition, the DHI% values did not differ significantly between the injured and single hydrogel groups after 4 and 8 weeks (Figure 7B–D). MRI analysis of T2‐weighted signal images could detect the water content of the IVD, reflecting IVD regeneration. As shown in Figure 7E, the siP65@G5‐PBA@Gel and the NP cell co‐delivery groups exhibited greater signal intensity of the T2‐weighted MRI images than the injured and Gel groups at both 4 and 8 weeks after surgery, suggesting a higher water content in the NP. In addition, siP65@G5‐PBA@Gel combined with cell therapy could further enhance the signal intensity at 8 weeks, while the siP65@G5‐PBA@Gel showed a decreasing tendency. The quantitative MRI grade results were consistent with the MRI images (Figure 7F). These results suggest that hydrogel injection alone had a limited therapeutic effect and that the gene drug‐loaded hydrogel could significantly delay the decrease in disc height and water content. The hydrogel co‐delivered gene‐drug and NP cells could not only reverse the reduced disc height but also maintain the tissue water content for a long duration.

Next, histological evaluation was performed to assess IVD regeneration. As shown in Figure 7G,H, H&E, Safranin O, and Fast Green staining revealed massive cell infiltration and an extremely disordered disc structure in the injured (Group II) and Gel injection groups (Group III) after both 4 and 8 weeks. SiP65@G5‐PBA@Gel (Group IV) exhibited slightly reduced NP areas at 4 weeks, which became more severe at 8 weeks. In contrast, the co‐delivery group (Group V) exhibited more intact NP and better‐organized annulus fibrosus tissues than the other groups, except for the control group at 4 weeks, and was similar to the control group at 8 weeks (group I). Additionally, the quantitative histological grade in the co‐delivery group was lower than those in the injured, Gel, and siP65@G5‐PBA@Gel groups after 4 and 8 weeks (Figure 7I). Immunohistochemistry (IHC), measuring the content of ECM constituents (Col2a1 and ACAN), had comparable results to the H&E, safranin O, and fast green staining. Specifically, at both 4 and 8 weeks, the injured and Gel groups exhibited few Col2a1 and ACAN staining areas. The co‐delivery group showed higher Col2a1 and ACAN expression levels than the siP65@G5‐PBA@Gel group after 4 weeks, but lower levels than the control group; the levels recovered at 8 weeks and were similar to the control group (Figure 7J–L). Together, these results strongly suggest that the gene‐drug‐loaded hydrogel exhibited excellent effects in delaying the process of IVDD and has great potential to promote IVD regeneration when combined with cell therapy.

**Figure 8:**
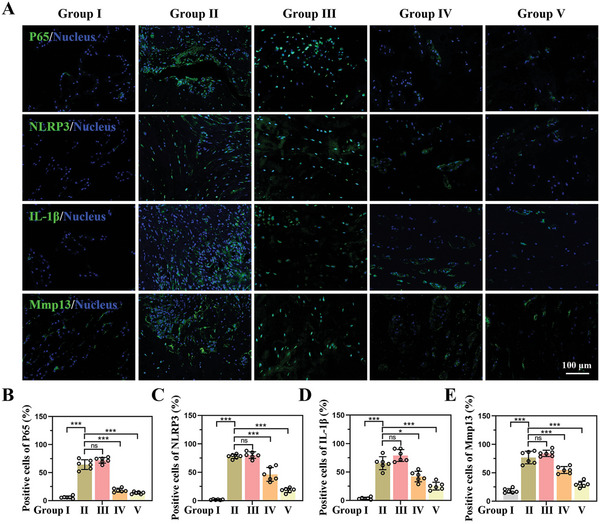
Underlying mechanism of the siP65@G5‐PBA@Gel combined with cell therapy for IVD regeneration in vivo. A) IF images of the P65, NLRP3, IL‐1*β*, and Mmp13 expression in different groups at 8 weeks postoperative (scale bar = 100 µm). B–E) Quantitative positive cells of P65, NLRP3, IL‐1*β*, and Mmp13 analyzed by Image J (*n* = 6 discs). The values presented are the mean ± SD. * *p* < 0.05; *** *p* < 0.001; ns, not statistically significant. Statistical significance was assessed by one‐way ANOVA with a post‐hoc Tukey test for (B–E).

Finally, the mechanism of siP65@G5‐PBA@Gel combined with cell therapy for IVD regeneration in vivo was closely explored. The levels of P65, NLRP3, IL‐1*β*, and Mmp13 in the different groups at 8 weeks after surgery were determined by immunofluorescence (IF). The IF images showed that compared with the control group, the P65, NLRP3, IL‐1*β*, and Mmp13 levels in NP areas of the injured and gel groups were significantly increased, which could be attenuated in both the siP65@G5‐PBA@Gel and NP cells co‐delivery groups (**Figure** **8**). The efficient and prolonged gene silencing effect of the gene‐drug‐loaded hydrogel in vivo was thus fully confirmed, and the results are consistent with the results in vitro. Through accurate and long‐term P65/NLRP3 signaling pathway inhibition, siP65@G5‐PBA@Gel can effectively regulate the inflammatory microenvironment, reverse the course of IVDD, and achieve successful IVD regeneration when combined with cell therapy.

## 3 Conclusion

In summary, a novel, injectable, self‐healing, adhesive, hemostatic, antibacterial, and biocompatible multifunctional hydrogel was prepared based on multi‐dynamic bonds for the spatiotemporal delivery of siRNA@G5‐PBA nanocomplexes targeting the P65/NLRP3 inflammatory signaling pathway to achieve precise, efficient, and sustained gene therapy. Specifically, localized gene‐drug delivery and temporally microenvironment‐responsive gene‐drug release resulted in the spatiotemporal regulation of cellular gene expression. In vitro release of a gene‐drug from the multifunctional hydrogel could significantly silence P65/NLRP3 pathway and inhibit inflammatory storms and the subsequent degeneration of NP cells induced by LPS. In vivo experiments also verified that the siRNA@G5‐PBA@Gel could successfully attenuate the inflammatory response through sustained inhibition of the P65 expression. In addition, the as‐fabricated hydrogel system showed excellent cellular biocompatibility and was an excellent cell encapsulator. By combining gene therapy with cell therapy, dramatically promoted regeneration of IVD was achieved. This high‐performance hydrogel system has promising clinical application prospects for minimally invasive therapy of IVDD and other inflammation‐related diseases. This study may provide new insights into the development of highly efficient drug delivery systems.

## 4 Experimental Section

Methods and any associated references are available in the Supporting Information.

## Conflict of Interest

The authors declare no conflict of interest.

## Supporting information

Supporting Information

Supplemental Video 1

Supplemental Video 2

## Data Availability

Research data are not shared.
