# Supplementary material for: High‐Performance Multi‐Dynamic Bond Cross‐Linked Hydrogel with Spatiotemporal siRNA Delivery for Gene–Cell Combination Therapy of Intervertebral Disc Degeneration
Source: Adv Sci (Weinh). 2023 Apr 20;10(17):2206306. doi: 10.1002/advs.202206306 (PMC10265076; doi:10.1002/advs.202206306)
Supplement: Supplementary file 1 — Supporting Information [file ADVS-10-2206306-s003.pdf]

ADVANCED SCIENCE
Open Access

Supporting Information

for *Adv. Sci.*, DOI 10.1002/advs.202206306

High-Performance Multi-Dynamic Bond Cross-Linked Hydrogel with Spatiotemporal siRNA Delivery for Gene–Cell Combination Therapy of Intervertebral Disc Degeneration

Jiaxin Chen, Haifeng Zhu, Jiechao Xia, Yutao Zhu, Chen Xia, Zehui Hu, Yang Jin, Ji Wang, Yong He, Jiayong Dai and Zhijun Hu**

Supporting Information

High-Performance Multi-Dynamic Bond Cross-Linked Hydrogel with Spatiotemporal siRNA Delivery for Gene–Cell Combination Therapy of Intervertebral Disc Degeneration

Jiaxin Chen [b,†], Haifeng Zhu [a,†], Jiechao Xia [a,†], Yutao Zhu [a], Chen Xia [c], Zehui Hu [a], Yang Jin [a], Ji Wang [b], Yong He [d], Jiayong Dai [a,*], and Zhijun Hu [a,*]

[a] Key Laboratory of Musculoskeletal System Degeneration, Regeneration Translational Research of Zhejiang Province, Department of Orthopaedic Surgery, Sir Run Run Shaw Hospital, Zhejiang University School of Medicine, Hangzhou 310016, China.

[b] Center for Plastic & Reconstructive Surgery, Department of Plastic & Reconstructive Surgery, Zhejiang Provincial People's Hospital, Affiliated People's Hospital, Hangzhou Medical College, Hangzhou 310014, China.

[c] Department of Orthopedic Surgery, Zhejiang Provincial People's Hospital, Affiliated People's Hospital, Hangzhou Medical College, Hangzhou 310014, China.

[d] State Key Laboratory of Fluid Power and Mechatronic Systems, College of Mechanical Engineering, Zhejiang University, Hangzhou 310027, China.

E-mail: daijy@zju.edu.cn; hzjspine@zju.edu.cn.

[†] These authors contributed equally to this work.

Experimental Section

Materials and Reagents

Amine-terminated G5 PAMAM was purchased from Weihai Chenyuan Molecular New Material Co., Ltd. (Shandong, China). 4-(Bromomethyl)phenylboronic acid (PBA), gelatin, 1-(3-Dimethylaminopropyl)-3-ethylcarbodiimide hydrochloride (EDC), N-hydroxysuccinimide (NHS), 1-hydroxybenzotriazole monohydrate (HOBt), adipic acid dihydrazide (ADH), hydro-caffeic acid (HCA), $NaIO_4$, ethylene glycol, and Girard reagent T were purchased from Aladdin (Shanghai, China). Dextran (100–200 kDa) was obtained from Yuanye Bio-Technology Co., Ltd. (Shanghai, China). Dulbecco's modified Eagle's medium, Ham's F-12 medium (DMEM/F12), and fetal bovine serum (FBS) were purchased from Gibco (Carlsbad, CA, USA). siRNA, FAM, or Cy5 labeled siRNA were synthesized by RiboBio Co., Ltd. (Guangzhou, China), and the siRNA sequences are summarized in **Table S1, Supporting Information**. Lipofectamine 3000 was purchased from Invitrogen (Carlsbad, California). Antibodies of NLRP3, Mmp3 and Mmp13, P-P65 and P65, Col2a1, ACAN, and β-actin were purchased from Abcam (Cambridge, UK), Cell Signaling (Shanghai, China), and Proteintech (Wuhan, China), respectively.

Synthesis and Characterization of G5-PBA

PBA-modified G5 PAMAM (G5-PBA) was synthesized as previously described.[1] Briefly, PBA (128 μmol) was reacted with dendrimer at a molar ratio of 128:1 in dimethyl sulfoxide (DMSO) at 70 °C for 48 h, followed by extensive dialysis against dimethyl sulfoxide and distilled water. Purified G5-PBA was then freeze-dried and characterized by proton nuclear magnetic resonance spectroscopy (^1H NMR) in D_2O (AVANCE III 400 Hz, Switzerland).

Preparation and Characterization of siRNA@G5-PBA Complexes

G5-PBA and 200 pmol siRNA dissolved in diethyl pyrocarbonate-treated water were mixed at different N/P ratios and cultured at room temperature for 10 min to prepare the gene transfection complexes. The particle sizes and zeta potentials of the siRNA@G5-PBA complexes were measured using a Zetasizer Nano-ZS (Malvern, UK). The morphology of the siRNA@G5-PBA complexes was observed by TEM (HT-7700, Japan). The siRNA binding and RNase degradation prevention capacity of G5-PBA were determined by agarose gel electrophoresis.

Extraction and Culturing of NP cells

Sprague-Dawley rats were used for rat NP cell isolation; the protocol was reported in a previous study.[2] Briefly, Gel-like NP tissues separated from the lumbar discs using a dissecting microscope were cut into small pieces and digested with 0.2% type II collagenase for 4 h at 37 °C. The suspension was cultured in DMEM/F12 containing 10% fetal bovine serum (FBS) and antibiotics at 37 °C in 5% CO_2. The culture medium was changed every three days and the cells were expanded through two passages for further study.

Transfection Efficacy and Cytotoxicity of siRNA@G5-PBA Complexes *in Vitro*

Rat NP cells (5×10^4) were plated in 24-well plates and incubated for 24 h. Different N/P ratios of the FAM-siRNA (200 pmol)@G5-PBA complexes were prepared. The cells were incubated with the complexes for 6 h and observed under a fluorescence microscope (IX83-FV3000, Japan). After co-culturing for 24 h, 10% (v/v) Cell Counting Kit-8 (CCK-8) solution in DMEM/F12 was added to each well and incubated at 37 °C for 2 h to assess the toxicity. The optical density of each well was measured at 450 nm using a microplate reader

(Molecular Devices, USA). The optimal N/P ratio of the FAM-labeled complexes was co-cultured with rat NP cells for 6 h, and the transfection efficiency was accurately analyzed by flow cytometry (BD FACSCANTO II, USA) using excitation and emission wavelengths of 492 and 518 nm, respectively. For the gene silencing efficiency measurement, siP65@G5-PBA was incubated with rat NP cells for 6 h and then further cultured for 48 h with medium. The protein expression of P65 was tested using western blot (WB) tests. Cells transfected with Lipofectamine 3000 were used as positive controls.

Synthesis and Characterization of OG

DEX (4 g) was dissolved in deionized water at a concentration of 2% (w/v), and 4 g $NaIO_4$ was added to the solution and reacted in a dark environment for 6 h. Subsequently, 1 mL of ethylene glycol was added and stirred for 1 h to terminate the oxidation reaction. The solution was dialyzed against deionized water for three days (MWCO 8–14 kDa) and freeze-dried to obtain oxidized dextran (ODEX).[3] Next, 1 g ODEX was dissolved in 100 mL deionized water, 1 g Gillard reagent T was added, and the mixture was stirred at room temperature for 1 h with the pH value adjusted to 5. After dialysis and freeze-drying, OG was obtained. The chemical structures of ODEX and OG were identified using Fourier transform infrared spectroscopy (FTIR; VERTEX70, Germany) and ^{1}H NMR.

Synthesis and Characterization of GCA

Gelatin (1 g) was dissolved in 100 mL of deionized water, and 364 mg HCA, 382 mg EDC, and 230 mg NHS were added. After adjusting the pH to 5.0, the solution was reacted overnight at room temperature under nitrogen. The resulting solution was purified in a dialysis tube (MWCO 8–14 kDa) against deionized water for 3 d and lyophilized to obtain

catechol-coupled gelatin (GC).[4]

Next, 1 g GC was dissolved in 100 mL of deionized water, and 1.1 g ADH was added with stirring. Then, 0.23 g of EDC dissolved in 10 mL of deionized water and 0.23 g HOBt dissolved in 10 mL of DMSO were added dropwise. Subsequently, the pH of the mixed solution was maintained at 5.0, and the reaction was maintained overnight. The resulting products were dialyzed for three days and freeze-dried to obtain GCA.[5]

The chemical structure of the GCA was identified using ^{1}H NMR. The grafting rate of catechol in GC was calculated by measuring the absorbance at 280 nm with a UV-vis spectrophotometer (UV-3150, Japan),[6] and the grafting rate of ADH in GCA was tested using the TNBS assay.[7]

Preparation of the Multifunctional Hydrogels (OG/GCA)

The prepared OG polymer was dissolved in PBS at concentrations of 5%, 8.5% and 12% (w/v). The synthetic GCA monomer was dissolved in PBS at concentrations of 10% and 15%. Equal volumes of different concentrations of OG and GCA polymers were mixed at room temperature to prepare the hydrogels, which were denoted as OG/GCA1 (5% OG and 10% GCA), OG/GCA2 (8.5% OG and 10% GCA), OG/GCA3 (8.5% OG and 15% GCA), and OG/GCA4 (12% OG and 15% GCA).

Gelation Time

The gelation time of the hydrogels was investigated *via* the vial-tilting method.[8] Briefly, solutions of OG and GCA at varying concentrations were mixed at equal volumes in vials. The sol-gel transition time was tested by inverting the vial at 37 °C until no fluidity was observed (n = 3).

Rheological Tests

The rheological behavior of the hydrogels was determined with an Anton Paar MCR302 rheometer using a parallel plate with a diameter of 25 mm and a gap of 1 mm at 37 °C. The measured rheological parameters were the storage modulus (G′) and loss modulus (G″). First, frequency sweep tests were performed between 0.01 and 10 Hz using a constant strain (1%), and the average G′ at 1 Hz was calculated to assess the rheological mechanical properties of the hydrogels (n = 3). Then, the shear-thinning properties of the hydrogels were evaluated using a shear rate sweep at a fixed frequency of 1 Hz with shear rates ranging from 0.1 to 1000 s^{-1}. Subsequently, a strain amplitude sweep test (strain% (γ) = 0.1%–1000%) was performed to obtain the critical strain point. Finally, the capacity of the hydrogels to recover from strain deformation was measured by repeatedly subjecting the hydrogels to an oscillating strain of 1% (200 s for each interval) and 500% (100 s for each interval) at a frequency of 1 Hz.

Compression Tests

The hydrogel samples were fabricated into cylindrical shapes (8 mm diameter × 8 mm height) for compression testing. Compressive stress–strain measurements were performed using a universal testing machine (Zwick/Roell Z020, Germany). The compressive strain rate was 1 mm/min, and the elasticity modulus was calculated in the strain range of 10%–20% (n = 4).

Swelling Tests

Lyophilized hydrogels (400 µL) were weighed (W_0) and then incubated in 2 mL PBS solution (pH = 7.4) at 37 °C (n = 3). At a predetermined time point, the swollen wet

hydrogels, dried superficially with a filter paper, were weighed and designated as W_t. The swelling percentage of the hydrogels was calculated using the following equation: swelling percentage (%) = $(W_t - W_0)/W_0 \times 100\%$.

In Vitro Degradation Tests

The *in vitro* degradation of the hydrogels under physiological conditions was studied by incubating freshly prepared hydrogel samples (400 μL) in PBS at 37 °C and a speed of 100 rpm (n = 3). At day 7, 14, 21, and 28, the samples were then weighed (W_t), and the degradation of the hydrogels was calculated using the following formula: weight remaining (%) = $W_t/W_0 \times 100\%$.

Scanning Electron Microscopy (SEM) Evaluation

Cross-sections of different freeze-dried hydrogels were sprayed with gold and observed by SEM (Nova Nano 450, USA) to assess the microscopic morphology of the surfaces. Image J software was employed to measure the porosity of hydrogel samples (n = 3).

Macroscopic Assessment

To evaluate their injectability, the OG and GCA solutions were added to different channels of a syringe, mixed, and injected through a dual-channel injector. The prepared hydrogels were also added into a syringe and injected through a 21G needle.

Two pieces of hydrogels artificially dyed with rhodamine B and methylene blue were cut in half. Subsequently, two semicircles of different colors were placed together for 30 min at room temperature to observe the self-healing behavior.

The stained hydrogels were placed into molds of different shapes to demonstrate their ability to fill irregular defects.

To illustrate the macroscopic wet tissue adhesive property, hydrogels adhering to various wet tissues, including heart, liver, spleen, lung, kidney, and IVDs were photographed. Besides, the hydrogels adhering to porcine skins were then underwent underwater placed and flushed by running water, which were recorded.

Adhesive Strength Tests of the OG/GCA Hydrogels

Lap shear tests were performed to quantitatively measure the wet tissue adhesive ability of the hydrogels using fresh porcine skin, as described in a previous study.[9] Briefly, hydrogels (400 μL) were applied to the surface between two porcine skins with an adhesive area of approximately 10 mm × 10 mm, and then the tissues were maintained at room temperature for 10 min. The adhesion properties were tested using a universal testing machine with a 100 N load cell at a strain rate of 1 mm/min. The adhesive strength was calculated as the maximum stress divided by the bonded area (n = 3).

Antibacterial Activity Evaluation

Staphylococcus aureus (*S. aureus*), *Escherichia coli* (*E. coli*), and carbapenem-resistant *Klebsiella pneumoniae* (CRKP) were used to test the antibacterial activities of the hydrogels. Hydrogel samples (400 μL) were immersed in 1 mL PBS buffer containing 10^4 CFU/mL bacteria and cultured for 12 h at 37 °C with shaking at 200 rpm. PBS containing 10^4 CFU/mL bacteria was used as the control group. Then, 10 μL from each group was spread onto Luria-Bertani (LB) plates and incubated for 12 h at 37 °C. Next, the number of colonies on the plates was recorded, and the antibacterial ratio was calculated using the following equation: Antibacterial ratio (%) = $(C_0 - C_h)/C_0 \times 100\%$, where C_0 and C_h are the number of colonies in the control and hydrogel groups (n = 3), respectively. In addition, the bacterial

culture medium after co-cultivation with the OG/GCA hydrogel was centrifuged at 12000 rpm for 10 min, and the morphology and state of the bacteria were observed by SEM to further evaluate the antibacterial mechanism.

***In Vivo* Hemostatic Ability**

Rats with liver hemorrhage (Sprague-Dawley, four-week-old, male) were used to test the hemostatic potential of the OG/GCA hydrogels *in vivo*. Briefly, the rats were anesthetized with pentobarbital sodium and fixed to a 30° slanted operating board. The liver was then exposed by abdominal incision using surgical scissors, and a pre-weighed filter paper was placed under the liver. Next, the liver tip was cut off to form an open wound approximately 1.0 cm in length and quickly covered using hydrogels. The blood loss was recorded according to the weight of the filter paper. Untreated rats were used as the blank control group (n = 4).[10]

Biocompatibility Evaluation of the siRNA@G5-PBA@Gel

First, 200 pmol siRNA@G5-PBA complex was mixed with 200 µL GCA for 10 min at room temperature, and then mixed with 200 µL OG to form gene drug-loaded hydrogels (siRNA@G5-PBA@Gel). Rat NP cells were seeded at a density of 5×10^4 cells per well in 24-well plates and incubated for 24 h. The gel or siRNA@G5-PBA@Gel was transferred to cell culture inserts (8 µm pore size) and co-cultured with NP cells for 1 or 3 d. CCK-8 was used to detect the cytotoxicity of the hydrogels by measuring the absorbance at 450 nm using a microplate reader. To test the effect of hydrogels on cells in direct contact, 400 µL of gel or siRNA@G5-PBA@Gel was placed on the bottom of 24-well plates, and cells with a density of 1×10^5 cells per well were cultured on the surface of the hydrogels. On days three and

seven the samples were gently washed with PBS and then incubated with live/dead to measure the cell viability or stained with phalloidin to evaluate the morphology of the cell adhesion. Cells on the surface of the hydrogels were observed under a confocal microscope (Nikon A1 Ti, Japan).

The hemolysis assay was used to evaluate the blood compatibility of the hydrogels. Briefly, erythrocytes were obtained from rat blood (Sprague-Dawley, 8-week-old, male) by centrifugation at 1000 rpm for 5 min. PBS was then used to wash and dilute the obtained erythrocytes to a final concentration of 2% (v/v). Next, 400 μL of gel or siRNA@G5-PBA@Gel mixed with 1 mL erythrocyte stock was added to 24-well plates and shaken in an incubator at 37 °C for 1 h at a shaking speed of 100 rpm. 0.1% Triton X-100 was used as a positive control, whereas normal saline was used as the negative control. Next, the suspension was collected and centrifuged at 1000 rpm for 10 min. The absorbance was read at 540 nm using a microplate reader to calculate the hemolysis ratio using the following equation: Hemolysis ratio (%) = $(A_s – A_b)/(A_t – A_b) \times 100\%$, where A_s, A_b, and A_t represent the absorbance values of the supernatant of the hydrogels, normal saline, and Triton X-100 groups, respectively (n = 3).

To evaluate the *in vivo* host response of the hydrogels, rats (Sprague-Dawley, 8-week-old, male) were anesthetized with pentobarbital sodium, and 400 μL gel or siRNA@G5-PBA@Gel was injected subcutaneously at the back of the rats. At a preset time point, the skin and embedded hydrogels at the injection site were excised. The obtained samples were fixed in 4% paraformaldehyde (PFA), embedded in paraffin, sectioned, stained with hematoxylin and eosin (H&E), and observed under a high-quality microscope (Nikon

eclipse 80i, Japan). After eight weeks of subcutaneous embedding, the major organs (heart, liver, spleen, lung, and kidney) and blood were harvested. Histological changes in the organs were analyzed by H&E, whole blood samples were used for hematology analysis (TEK8500 VET), and indicators of the hepatic and kidney function of blood plasma samples were tested (n = 3).

***In Vitro* pH Response Release Test**

Cy5-labeled siRNA@G5-PBA nanocomplexes were prepared and loaded into the OG/GCA hydrogel (Cy5-siRNA@G5-PBA@Gel). Then, 2 mL PBS at pH 7.4 or 5.5 was incubated with Cy5-siRNA@G5-PBA@Gel in a shaker at 37 °C with a speed of 100 rpm. At the scheduled time, the supernatant was completely collected and replaced with fresh solution, and the release of siRNA@G5-PBA was quantified by Cytation3 (Bio-Tek, USA) using 649 and 680 nm excitation and emission wavelengths, respectively (n = 3).

***In Vivo* siRNA@G5-PBA Release Assessment**

Cy5-siRNA@G5-PBA@Gel samples (400 μL) were subcutaneously injected into the back of mice (C57BL/6, 6-week-old, male). Sustained and local release of siRNA@G5-PBA (649/680 nm) was monitored *via* Caliper IVIS Lumina II (CLS136341/F, USA) at a predetermined time (n = 3).

NP Cell Treatment

Gel or siP65@G5-PBA@Gel (400 μL) was placed into the bottom of 24-well plates. Then, 1×10^5 rat NP cells were seeded on the surface of the hydrogels and incubated with medium for 3 d. Lipopolysaccharide (LPS) (10 μg mL^{-1}) was then added to the medium for 2 d to induce inflammation and degeneration of the NP cells. The protein expression levels of P-P65,

NLRP3, IL-1β, TNF-α, Mmp3, Mmp13, and Col2a1 were detected *via* WB analysis.

Protein Extraction and WB Analysis

Treated NP cells were split using RIPA buffer containing proteinase and phosphatase inhibitors for 30 min, followed by centrifugation at 12000 rpm for 20 min to separate the supernatant. The protein concentration was measured using a BCA assay kit. Protein samples were separated by SDS-PAGE and electro-transferred onto nylidene difluoride PVDF membranes. The membranes were blocked with 5% skim milk and incubated with primary antibodies at 4 °C overnight, followed by incubation with the corresponding secondary antibodies for 1 h at room temperature. Then, proteins were detected using an ECL kit and visualized using a chemiluminescence imager system (SH-Cute523, China). Image J software (National Institutes of Health, USA) was used to quantify the gray values of the images, which were normalized to β-actin (n = 3).

Encapsulation of Rat NP Cells into siRNA@G5-PBA@Gel

Briefly, 5×10^5 rat NP cells were pre-suspended in 200 μL OG solution, and siRNA@G5-PBA complexes were pre-mixed with 200 μL GCA. Then, the two constituents were uniformly mixed to form bio-multifunctional hydrogels loaded with both the gene drug and cells. After encapsulation, the samples were cultivated in medium for 7 or 14 d, and live/dead staining was used to test the viability of the encapsulated NP cells.

Rat IVDD Model

A puncture-induced rat IVDD model was used to investigate the effects of hydrogels *in vivo*.[11] The surgical procedure has been described previously. Briefly, 20 rats (Sprague-Dawley, 8-week-old, male) were randomly divided into five groups (the positive

control, injured group, Gel group, siP65@G5-PBA@Gel group, and NP cells + siP65@G5-PBA@Gel group). Four rats underwent no surgical intervention as the positive controls and the remaining 16 rats underwent surgery as follows. The rats were anesthetized with pentobarbital sodium and palpated to determine the discs of the coccygeal (Co) vertebrae. Then, their discs (Co6/7, Co7/8, and Co8/9) were punctured using a 20G needle that passed through the whole disc, was rotated 180°, and held for 30 s. One week after the initial puncture, 20 μL of different hydrogels was injected into each disc through a 21G needle, except for the injured group without any treatment. The IVDs in each group were harvested after four or eight weeks for further experiments (six discs from two rats in each group at each time point).

Imaging Assessment

IVDs were imaged by micro-CT scanning (Siemens Inveon, Germany) using the protocol of 80 kV, 500 mA, and 14.97 mm isotropic resolution. The disc height was measured using Image J, and the disc height index (DHI%) was calculated using a previously described method (n = 6).[12]

MRI was performed using a 1.5T GE Sigma CV/I scanner (GE Healthcare, Japan) to acquire T2-weighted MR images in the median sagittal plane. MRI images were classified as grade I to IV based on assessment of the T2-weighted signal intensity (n = 6).[12] The imaging protocols were as follows: TR/TE of 3000/86, echo train length of 12, slice thickness of 2 mm, field of view of 10 cm, matrix size of 352 × 224, and number of excitations of 10.

Histological Analysis

IVDs were fixed in PFA, decalcified in EDTA, embedded in paraffin, and sliced for

histological evaluation. The sections were then stained with H&E, Safranin O, and Fast Green. Histological grading was performed as previously reported (n = 6).[12]

Immunohistochemistry (IHC) and Immunofluorescence (IF) Evaluations

For IHC, sections were placed in citrate sodium buffer overnight at 60 °C for antigen recovery, immersed in 3% H_2O_2 for 10 min, and blocked with 5% bovine serum albumin (BSA) at room temperature for 1 h. Subsequently, the sections were incubated with primary antibodies overnight at 4 °C, and then covered with biotin labeled secondary antibody at room temperature for 1 h; DAB was used to detect staining. Images were photographed using a microscope, and the relative positive areas were quantified using Image J (n = 6 discs).

For IF, after antigen recovery, the sections were permeabilized with 0.1% Triton X-100 for 30 min, followed by blocking with 5% BSA for 1 h at room temperature. The sections were then incubated with primary antibodies overnight at 4 °C. After washing three times with PBS, the sections were stained with Alexa Fluor 488 corresponding secondary antibodies and DAPI. Fluorescence images were captured using a fluorescence microscope. The number of positively stained cells was counted using Image J software (n = 6 discs).

Statistical Analysis

All of the statistical analyses were performed using SPSS 22.0 software (IBM, USA), and data are presented as the mean ± standard deviation (SD). The normality of data was first analyzed. For data conforming to the normal distribution, two-tailed Student’s t-test was used compare data between two independent groups, and one-way analysis of variance (ANOVA) with a post-hoc Tukey test was performed for the multiple comparisons. Data based on ordinal grading systems which did not conform to the normal distribution were analyzed by

the Kruskal-Wallis H test. * $P < 0.05$; ** $P < 0.01$; *** $P < 0.001$; ns, not statistically significant. Statistic differences were considered significantly at $P < 0.05$.

Table S1. Target siRNA sequence in this study.

siRNA	Target sequence
Rat-siP65	GCATCCAGACCAACAATAA

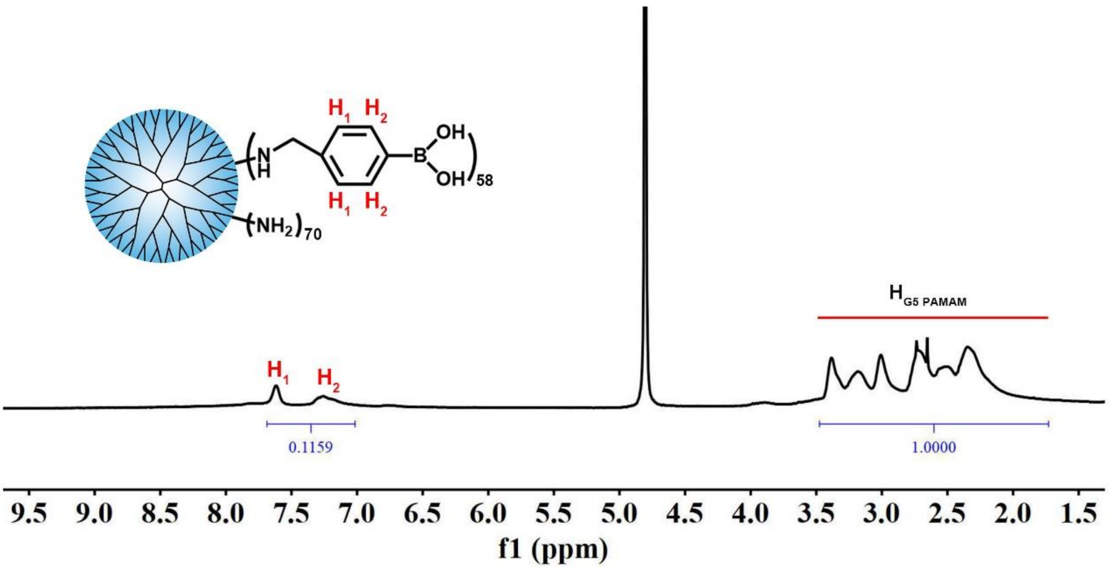

Figure S1. ^{1}H NMR spectrum of G5-PBA. The number of methylene protons on the G5 PAMAM dendrimer is 2016, and the number of aromatic protons on phenylboric acid is 4.[13] Based on the integrated areas, the average number of PBA conjugates was calculated to be 58.

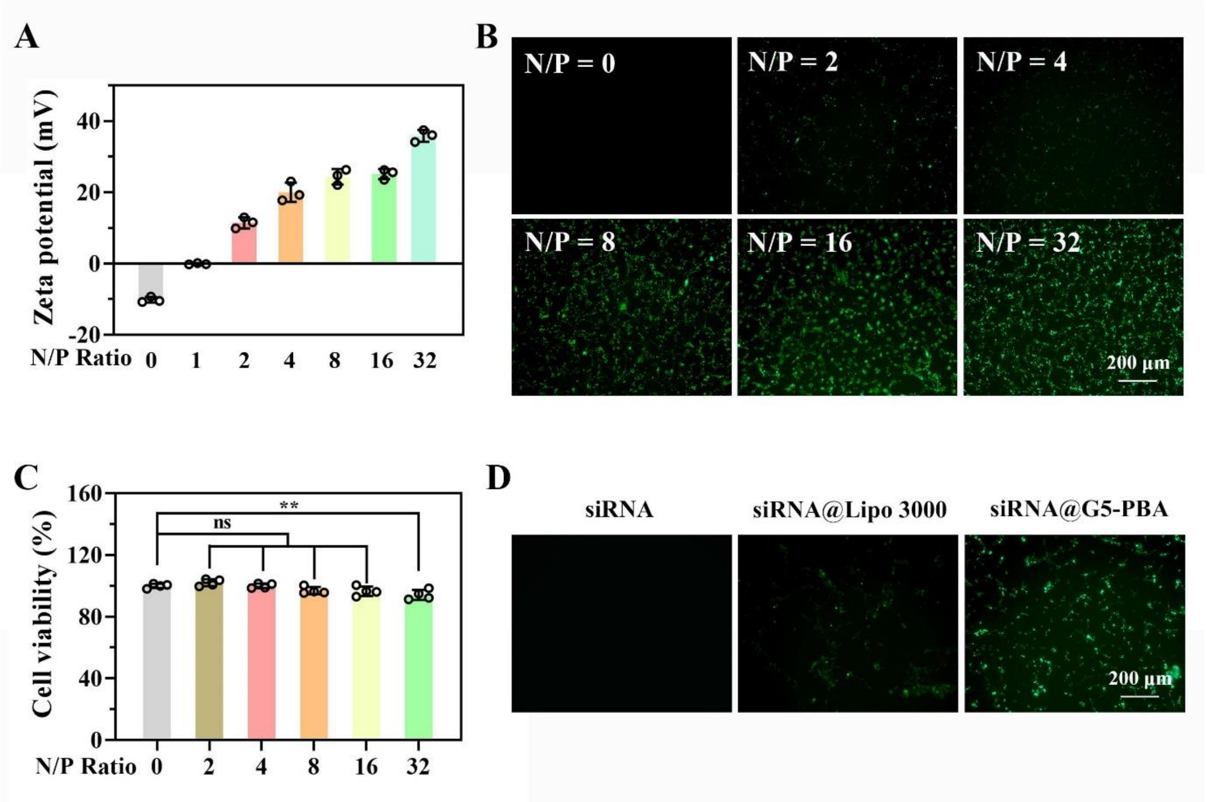

Figure S2. Characterization of siRNA@G5-PBA complexes. (**A**) Zeta potential analysis of complexes with different N/P ratios (n = 3). (**B**) Intracellular uptake of FAM-siRNA@G5-PBA complexes with different N/P ratios observed by fluorescence (scale bar = 200 nm). (**C**) Viability of rat NP cells after treatment with complexes having different N/P ratios for 24 h as tested by CCK-8 assay (n = 3). (**D**) Intracellular uptake of optimized FAM-siRNA@G5-PBA complexes compared to that delivered by Lipo300 transfection (scale

bar = 200 nm). The values presented are the mean ± SD. ** $P < 0.01$; ns, not statistically significant. Statistical significance was assessed by one-way ANOVA with a post-hoc Tukey test for **C**.

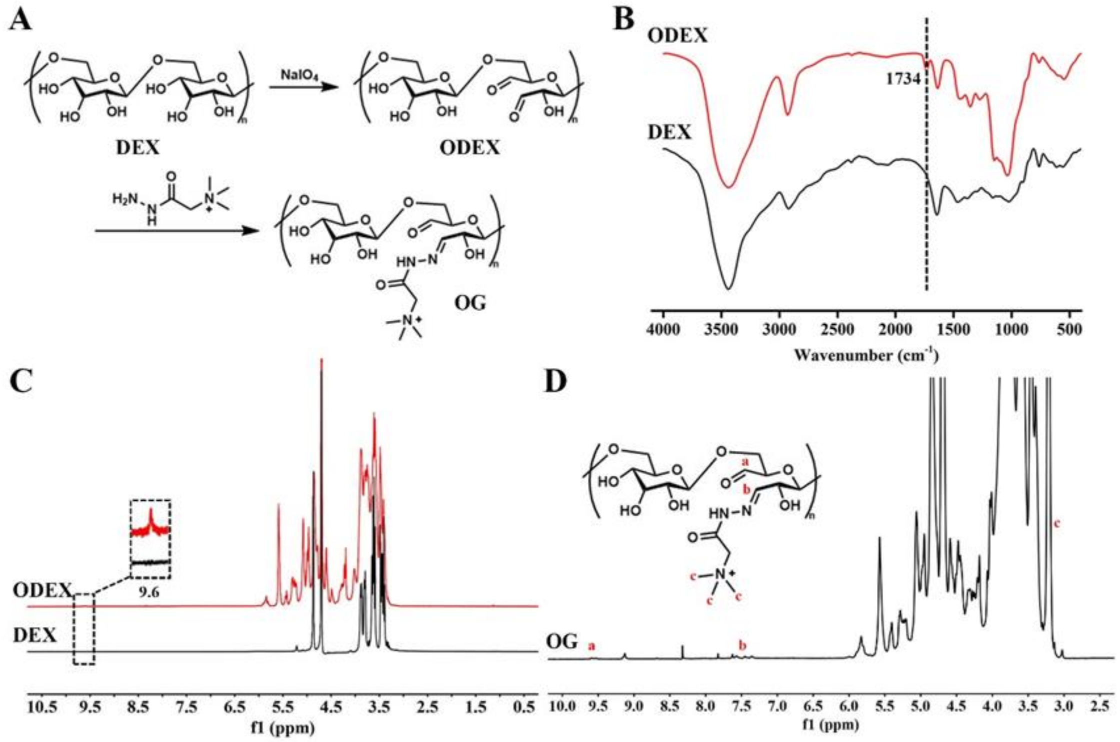

Figure S3. Synthesis and identification of OG. (**A**) Reaction route for the synthesis of OG. (**B**) FTIR spectra of ODEX and DEX. (**C**) ^{1}H NMR spectra of ODEX and DEX. (**D**) ^{1}H NMR analysis of OG.

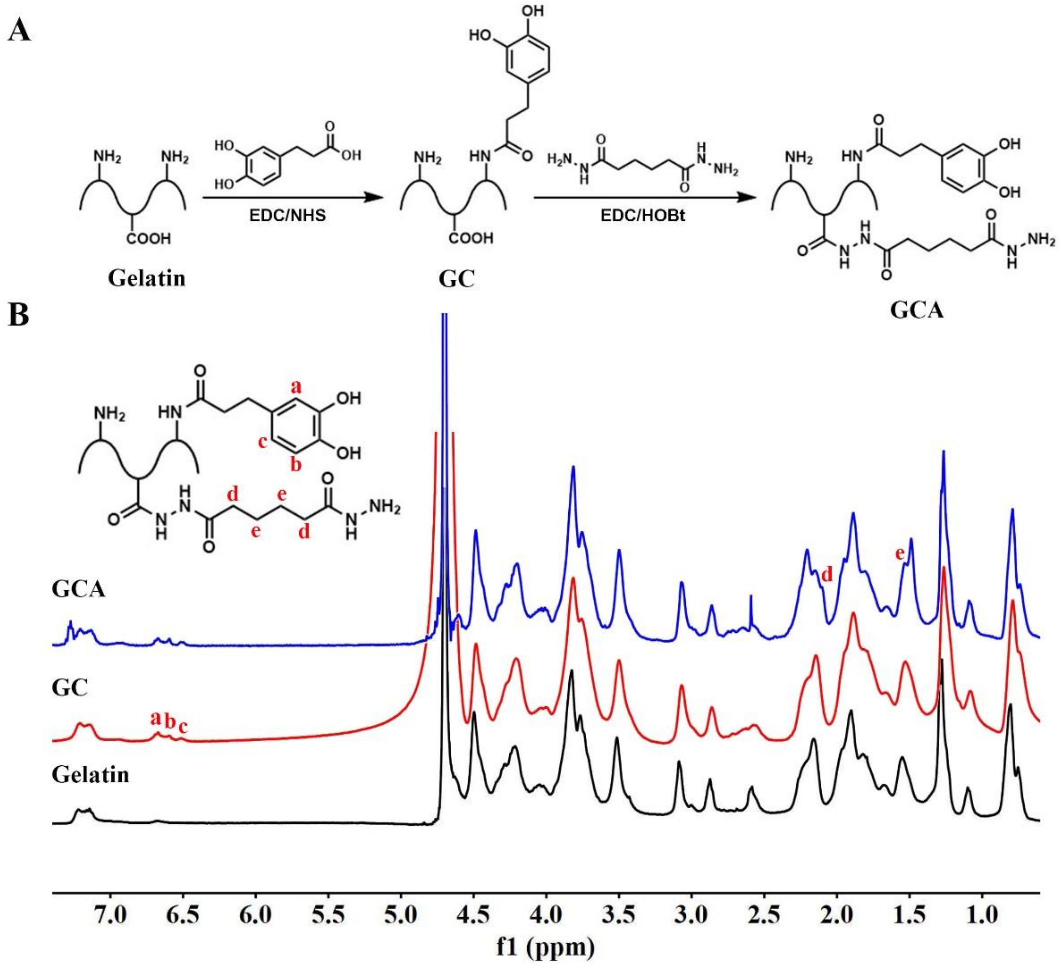

Figure S4. Synthesis and identification of GCA. (**A**) Synthetic route of GCA. (**B**) ^{1}H NMR spectra of unmodified gelatin, GC, and GCA.

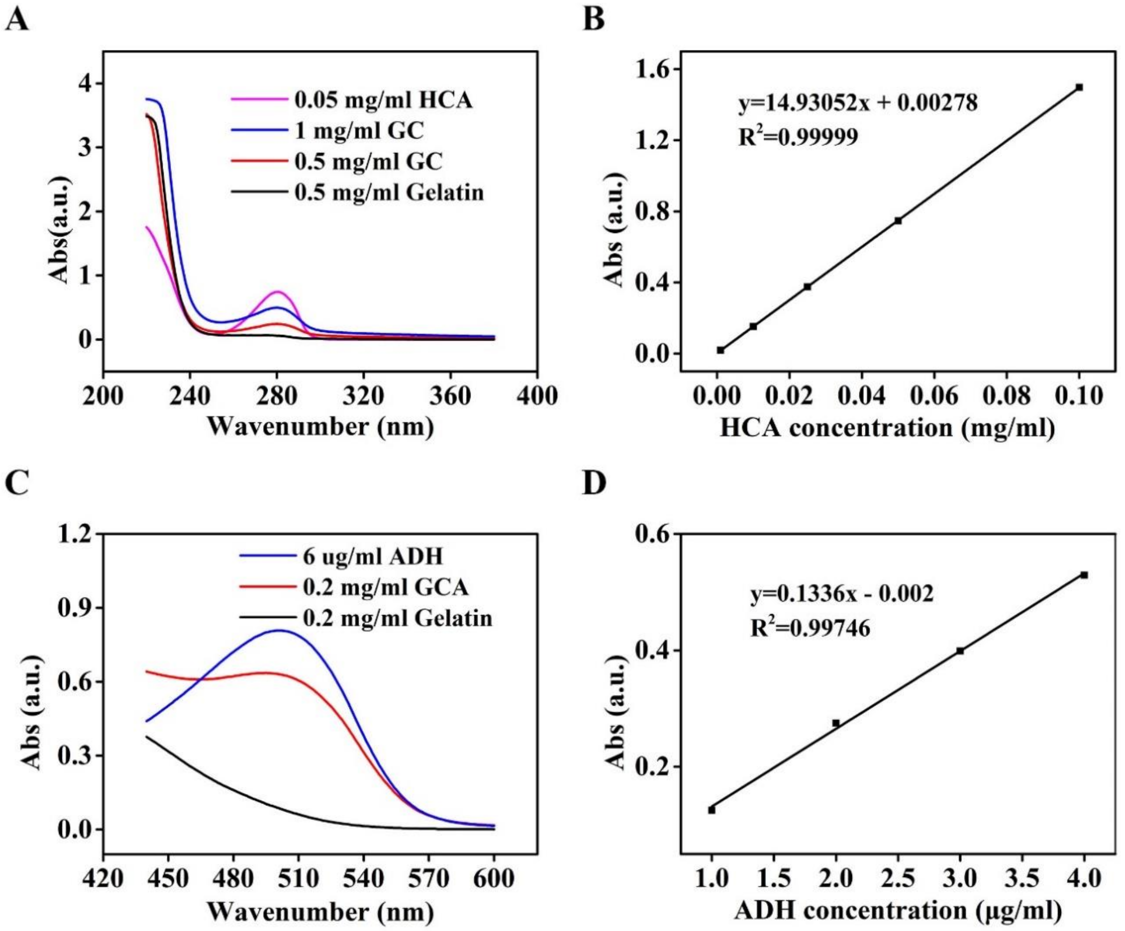

Figure S5. Assessment the grafting percent of HCA and ADH. (**A**) UV-vis spectra of HCA, gelatin, and GC. (**B**) Standard curve for HCA calculated from the UV-vis absorbance. (**C**) UV-vis spectra of ADH, gelatin, and GCA after reaction with TNBS. (**D**) Standard curves for ADH calculated from the UV-vis absorbance. According to the standard curve, the degrees of

substitution of HCA and ADH are 3.1% and 4.7%, respectively.

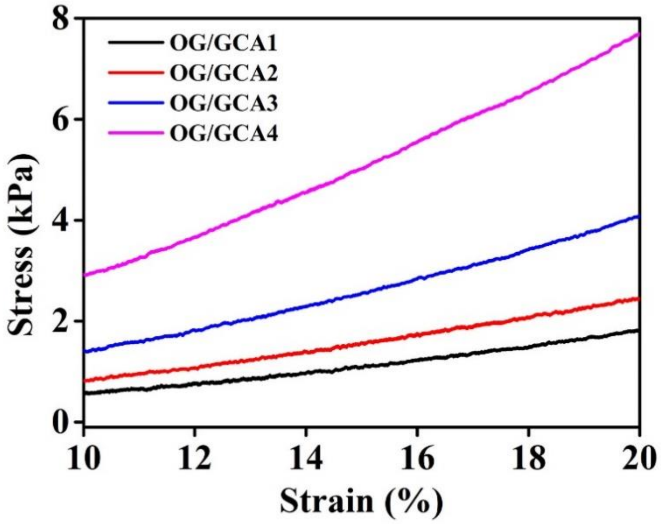

Figure S6. Compressive stress–strain curves of OG/GCA hydrogel with different concentrations (strain range of 10% to 20%). OG/GCA1: 5% OG and 10% GCA; OG/GCA2: 8.5% OG and 10% GCA; OG/GCA3: 8.5% OG and 15% GCA; OG/GCA4: 12% OG and 15% GCA.

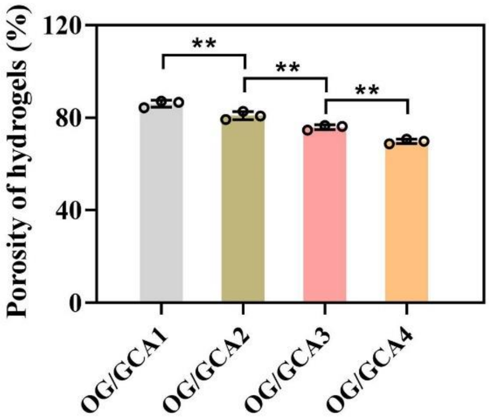

Figure S7. The porosity of hydrogels analyzed by Image J software (n = 3). OG/GCA1: 5% OG and 10% GCA; OG/GCA2: 8.5% OG and 10% GCA; OG/GCA3: 8.5% OG and 15% GCA; OG/GCA4: 12% OG and 15% GCA. The values presented are the mean ± SD. ** $P < 0.01$. Statistical significance was assessed by one-way ANOVA with a post-hoc Tukey test.

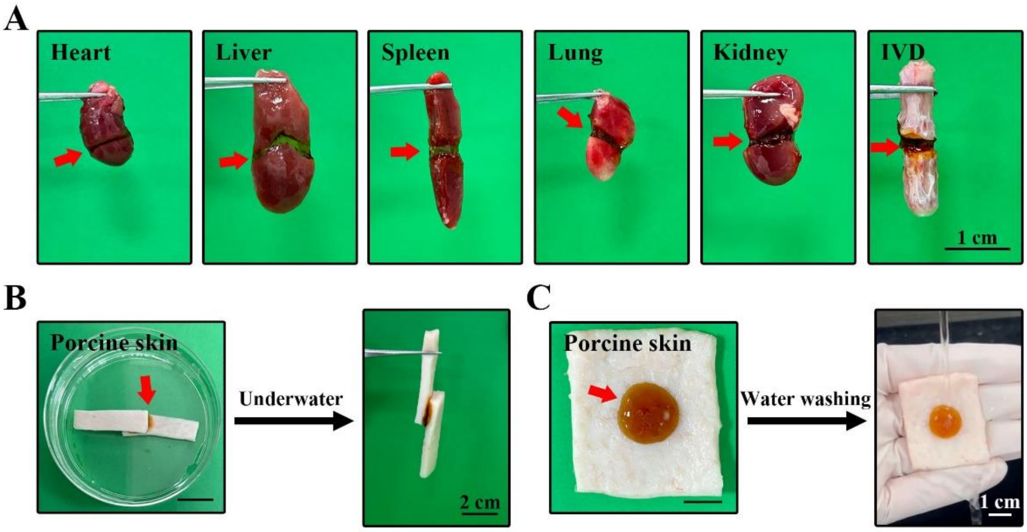

Figure S8. Macroscopic wet tissue adhesion property of the OG/GCA hydrogel. (**A**) Photographs of the hydrogel adhering to two pieces of fresh heart, liver, spleen, lung, kidney, and IVD tissues of rats (scale bar = 1 cm). (**B**) Photographs of the hydrogel adhering between two pieces of porcine skins after underwater placement. (scale bar = 1 cm). (**C**) Photographs of the hydrogel adhering to porcine skins underwent water washing. (scale bar = 1 cm). The red arrows indicate the adhesive hydrogel.

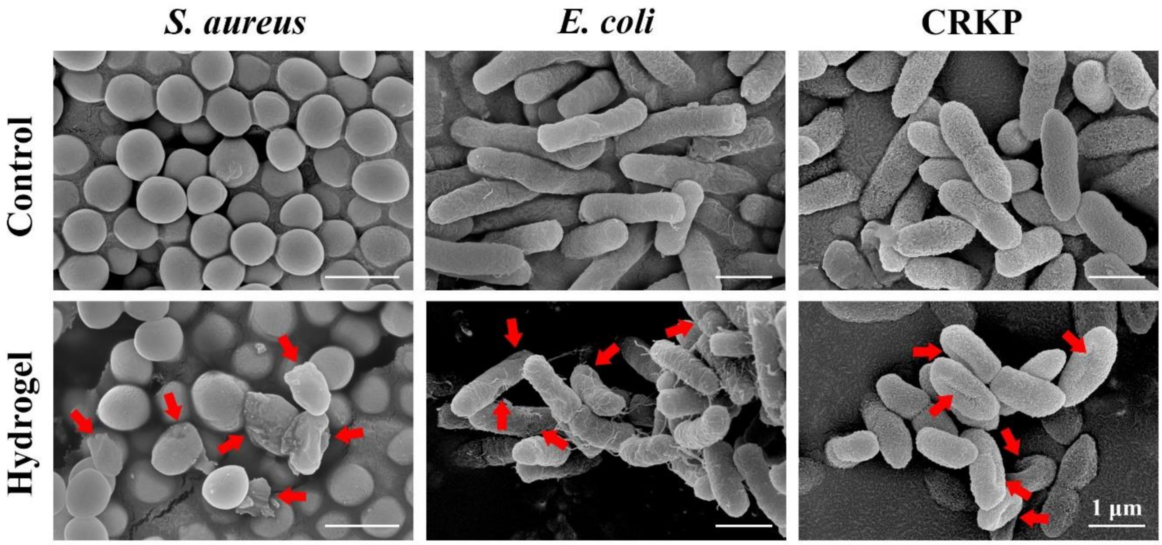

Figure S9. SEM analysis of the antibacterial mechanism of the OG/GCA hydrogel for *S. aureus*, *E. coli*, and CRKP *in vitro*. The red arrows indicate the wrinkled and damaged bacteria. Scale bar = 1 μm.

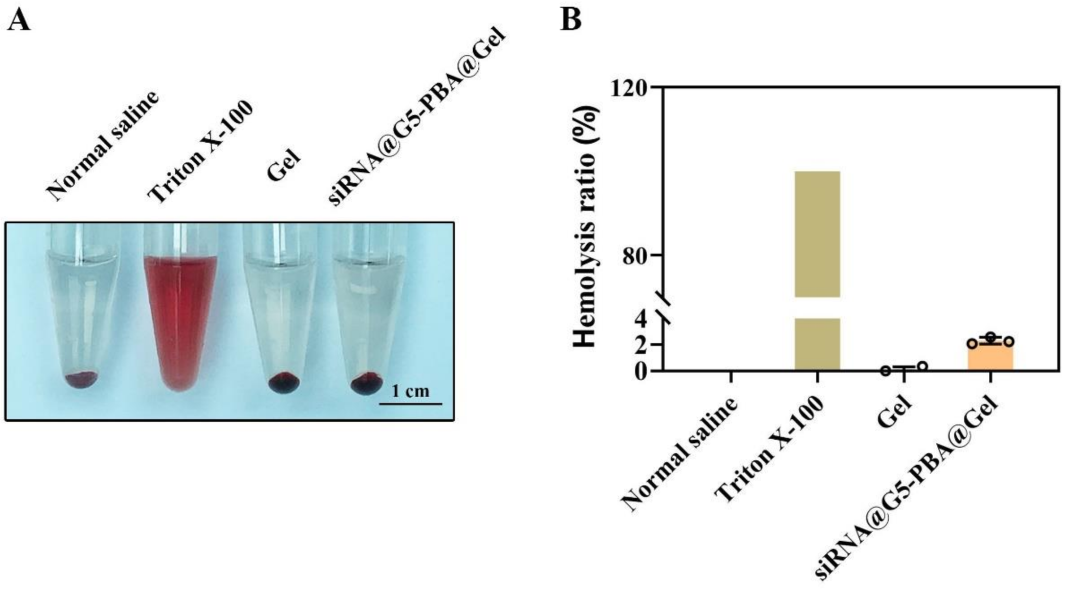

Figure S10. Hemocompatibility of the gene drug-loaded bio-multifunctional hydrogel. (**A**) Photographs (scale bar = 1 cm), and (**B**) quantitative results (n = 3) of the hemolytic test.

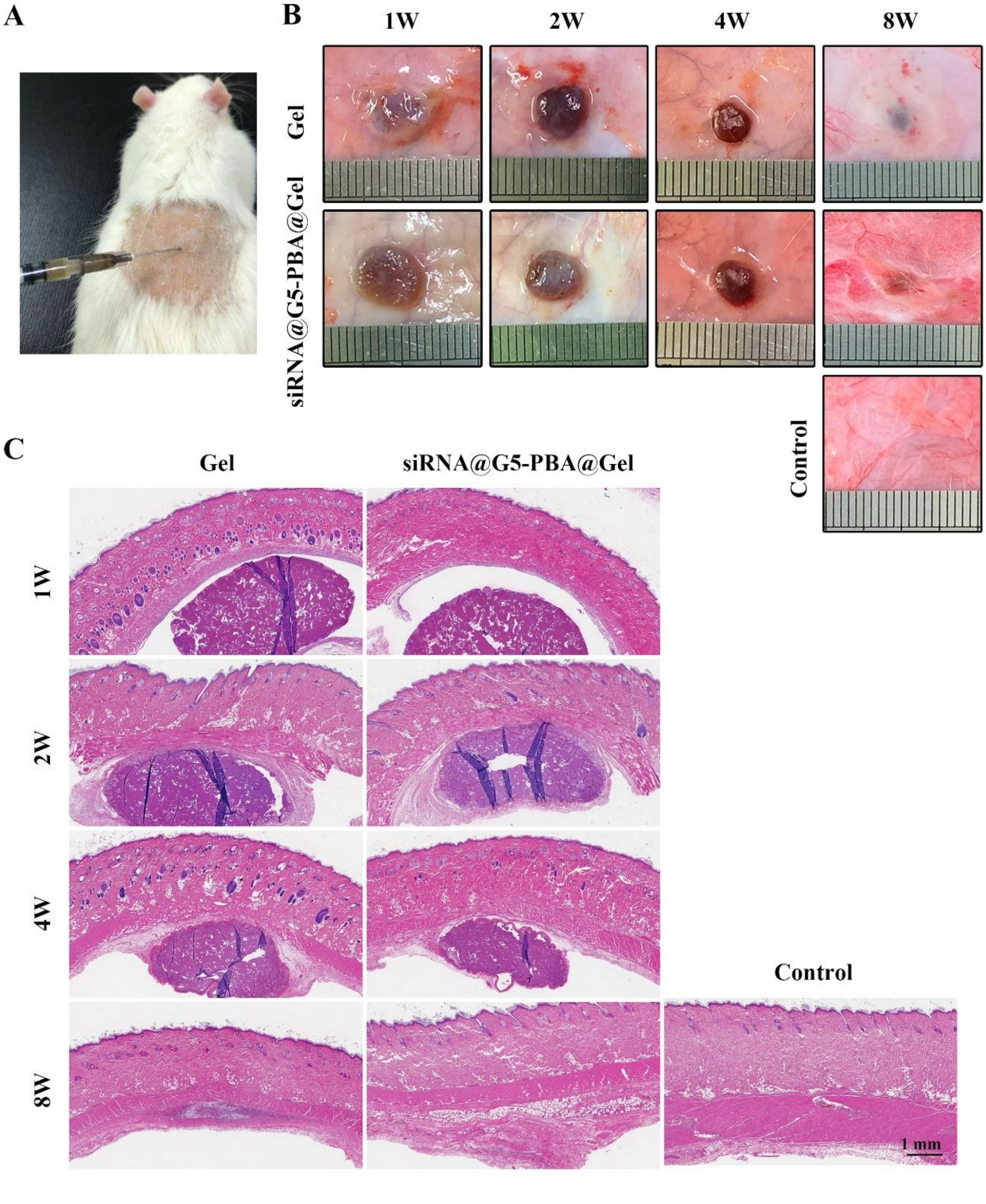

Figure S11. *In vivo* biodegradability and histocompatibility of hydrogels. (**A**) Photographs of

hydrogels injected subcutaneously into the backs of rats. (**B**) Photographs of the hydrogels and surrounding skin after 1, 2, 4, and 8 w. (**C**) H&E staining (scale bar = 1 mm).

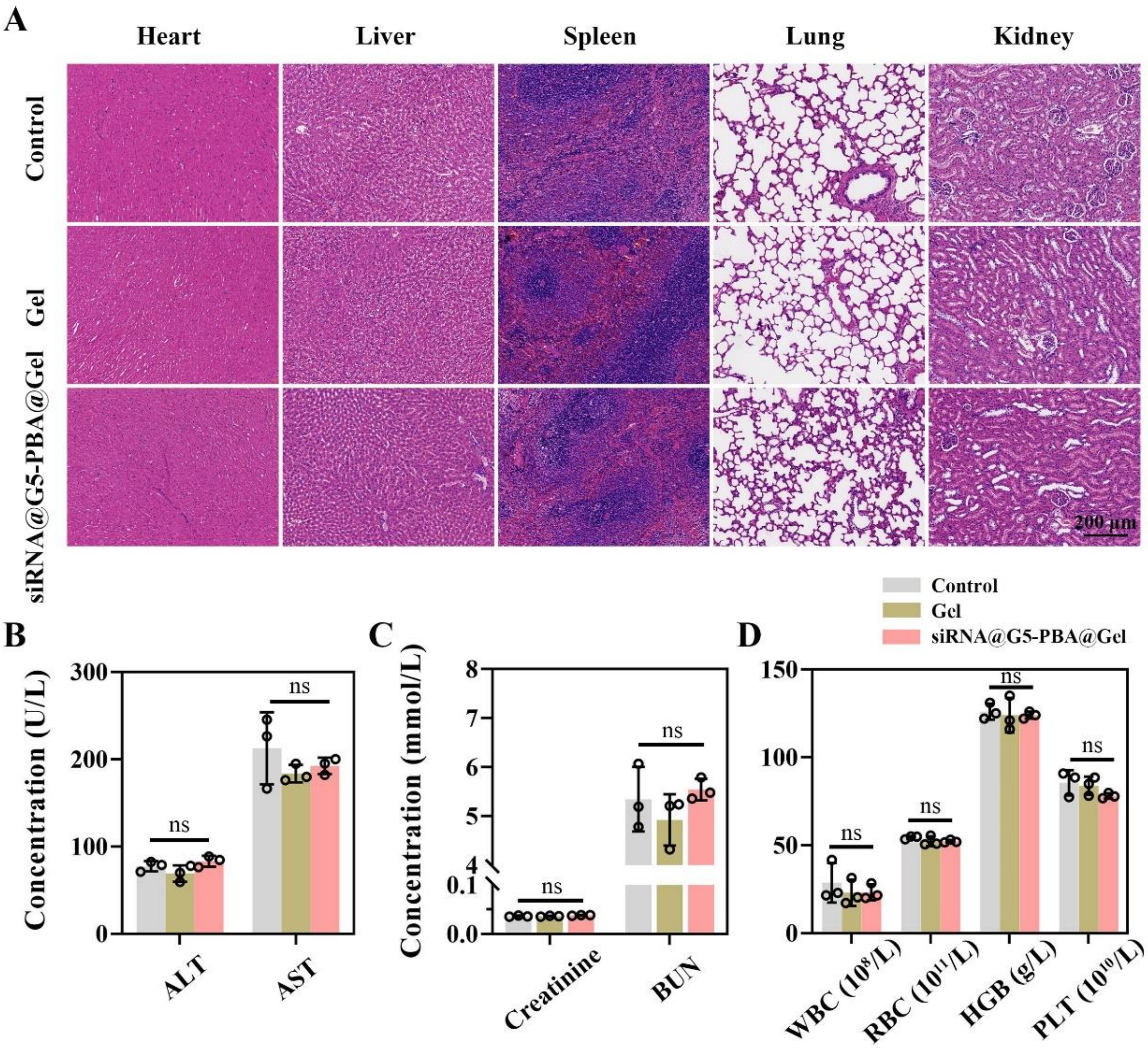

Figure S12. *In vivo* systemic toxicity assessment of hydrogels after eight weeks of implantation. (**A**) H&E staining of primary organs such as the heart, liver, spleen, lung, and kidney (scale bar = 200 μm). (**B**) Serum levels of liver function indicators including alanine transaminase (ALT) and aspartate transaminase (AST, n = 3). (**C**) Kidney function indicators including creatinine and blood urea nitrogen (BUN, n = 3). (**D**) Hematology analysis (n = 3).

The values presented are the mean ± SD; ns, not statistically significant. Statistical significance was assessed by one-way ANOVA with a post-hoc Tukey test for **B**, **C**, and **D**.

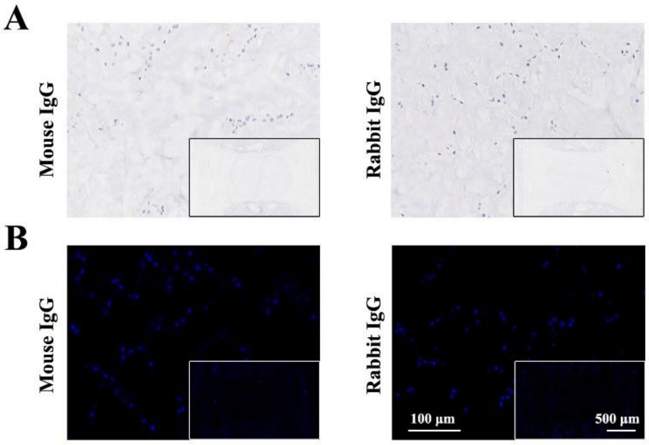

Figure S13. Negative controls of (**A**) IHC and (**B**) IF with mouse IgG or rabbit IgG substituted corresponding primary antibodies. Lack of positive staining indicates the antibody specificity. Scale bars were 500 μm for the embedded images and 100 μm for the magnified images, respectively.

References

[1] C. Liu, T. Wan, H. Wang, S. Zhang, Y. Ping, Y. Cheng, *Sci Adv* **2019**, 5, eaaw8922.

[2] S. Chen, X. Q. Fang, Q. Wang, S. W. Wang, Z. J. Hu, Z. J. Zhou, W. B. Xu, J. Y. Wang, A. Qin, S. W. Fan, *Lab Invest* **2016**, 96, 561.

[3] S. Guan, K. Zhang, L. Cui, J. Liang, J. Li, F. Guan, *Biomater Adv* **2022**, 133, 112604.

[4] W. Zhao, R. Xu, L. Zhang, Y. Zhang, Y. Wang, *Prog Org Coat* **2020**, 139, 105442.

[5] T. Hozumi, T. Kageyama, S. Ohta, J. Fukuda, T. Ito, *Biomacromolecules* **2018**, 19, 288.

[6] B. Yang, J. Song, Y. Jiang, M. Li, J. Wei, J. Qin, W. Peng, F. L. Lasaosa, Y. He, H. Mao, J. Yang, Z. Gu, *ACS Appl Mater Interfaces* **2020**, 12, 57782.

[7] T. Kageyama, T. Kakegawa, T. Osaki, J. Enomoto, T. Ito, T. Nittami, J. Fukuda, *Biofabrication* **2014**, 6, 025006.

[8] C. Arakawa, R. Ng, S. Tan, S. Kim, B. Wu, M. Lee, *J Tissue Eng Regen Med* **2017**, 11, 164.

[9] S. Yao, Y. Zhao, Y. Xu, B. Jin, M. Wang, C. Yu, Z. Guo, S. Jiang, R. Tang, X. Fang, S. Fan, *Adv Healthc Mater* **2022**, 11, 2200516.

[10] L. Xia, S. Wang, Z. Jiang, J. Chi, S. Yu, H. Li, Y. Zhang, L. Li, C. Zhou, W. Liu, B. Han, *Carbohydr Polym* **2021**, 264, 117965.

[11] a) W. Chen, H. Chen, D. Zheng, H. Zhang, L. Deng, W. Cui, Y. Zhang, H. A. Santos, H.

Shen, *Adv Sci (Weinh)* **2020**, 7, 1902099; b) Q. Zheng, H. Shen, Z. Tong, L. Cheng, Y. Xu, Z. Feng, S. Liao, X. Hu, Z. Pan, Z. Mao, Y. Wang, *Theranostics* **2021**, 11, 147.

[12] Y. Xu, Y. Gu, F. Cai, K. Xi, T. Xin, J. Tang, L. Wu, Z. Wang, F. Wang, L. Deng, C. L. Pereira, B. Sarmento, W. Cui, L. Chen, *Adv Funct Mater* **2020**, 30, 2006333.

[13] J. Lv, C. Liu, K. Lv, H. Wang, Y. Cheng, *Sci China Mater* **2019**, 63, 620.
